# Validation of an updated Associative Transcriptomics platform for the polyploid crop species *Brassica napus* by dissection of the genetic architecture of erucic acid and tocopherol isoform variation in seeds

**DOI:** 10.1111/tpj.13767

**Published:** 2017-12-02

**Authors:** Lenka Havlickova, Zhesi He, Lihong Wang, Swen Langer, Andrea L. Harper, Harjeevan Kaur, Martin R. Broadley, Vasilis Gegas, Ian Bancroft

**Affiliations:** ^1^ Department of Biology University of York Heslington York YO10 5DD UK; ^2^ Plant and Crop Sciences Division School of Biosciences University of Nottingham Sutton Bonington Campus Loughborough LE12 5RD UK; ^3^ Limagrain UK Ltd. Joseph Nickerson Research Centre Rothwell LN7 6DT UK

**Keywords:** association genetics, transcriptomics, *Brassica napus*, tocopherol, erucic acid

## Abstract

An updated platform was developed to underpin association genetics studies in the polyploid crop species *Brassica napus* (oilseed rape). Based on 1.92 × 10^12^ bases of leaf mRNAseq data, functional genotypes, comprising 355 536 single‐nucleotide polymorphism markers and transcript abundance were scored across a genetic diversity panel of 383 accessions using a transcriptome reference comprising 116 098 ordered coding DNA sequence (CDS) gene models. The use of the platform for Associative Transcriptomics was first tested by analysing the genetic architecture of variation in seed erucic acid content, as high‐erucic rapeseed oil is highly valued for a variety of applications in industry. Known loci were identified, along with a previously undetected minor‐effect locus. The platform was then used to analyse variation for the relative proportions of tocopherol (vitamin E) forms in seeds, and the validity of the most significant markers was assessed using a take‐one‐out approach. Furthermore, the analysis implicated expression variation of the gene *Bo2g050970.1*, an orthologue of *VTE4* (which encodes a γ‐tocopherol methyl transferase converting γ‐tocopherol into α‐tocopherol) associated with the observed trait variation. The establishment of the first full‐scale Associative Transcriptomics platform for *B. napus* enables rapid progress to be made towards an understanding of the genetic architecture of trait variation in this important species, and provides an exemplar for other crops.

## Significance Statement

The availability of a full‐scale association genetics platform for *Brassica napus*, based on functional genotypes, enables the genetic architecture of essentially any trait to be addressed in this important crop species.

## Introduction

As the demand for ever‐increasing crop productivity continues against the backdrop of climate change and diminishing resources, crop improvement has become an important driver for advances in genomic technologies in plants. A broad aim of crop science is the identification of the genetic bases for trait variation, including both the identification of beneficial alleles and the development of molecular markers to accelerate their introduction into elite germplasm. Genetic diversity panels, typically comprising past and current cultivars along with wild relatives, are usually available for crop species. Such panels represent ideal resources for genome‐wide association studies (GWAS), which exploit historical recombination between molecular markers and loci associated with trait variation. Where recombination between loci is observed proportionately less frequently than expected for unlinked loci (i.e. < 0.5), those loci are said to be in linkage disequilibrium (LD). The approach of identifying molecular markers in LD with loci associated with trait variation is an important tool used in human genetics studies, and has been applied successfully in several plant species (Garrigan and Hammer, 2006; Li *et al*., 2008; Atwell *et al*., 2010; Cockram *et al*., 2010; Tian *et al*., 2011; Zhao *et al*., 2011). The recent development of transcriptome‐based GWAS, including the technology termed Associative Transcriptomics (AT), in which both gene sequence variation and transcript abundance variation are used to identify associations with trait variation (Harper *et al*., 2012), greatly increases the range of crops to which GWAS approaches can be applied.

The Brassicaceae family includes *Arabidopsis thaliana*, the first plant for which a high‐quality genome sequence was available (AGI, 2000), and the Brassica crops. The diploid species *Brassica rapa* and *Brassica oleracea*, which contain the Brassica A and C genomes, respectively, are closely related, having shared a common ancestor only *c. *3.7 Mya (Inaba and Nishio, 2002). *Brassica napus* is an allopolyploid arising from the hybridization of these species (U. N. 1935), and the related (homoeologous) regions of the genomes are clearly discernible (Bancroft *et al*., 2015). A diverse range of *B. napus* crop types have been developed, including oilseed rape, fodders, leafy vegetables and root vegetables. Brassica species have been used extensively in genomics studies, because of their utility in studying the evolution of polyploid genomes (Song *et al*., 1995; O'Neill and Bancroft, 2000; Pires *et al*., 2004; Town *et al*., 2006; Yang *et al*., 2006; Cheung *et al*., 2009). A draft genome sequence has been obtained for *B. napus* (Chalhoub *et al*., 2014); however, at approximately 1.2 Gb, the genome of *B. napus* is relatively large. To address this problem, rapid and cost‐effective transcriptome‐based technologies, using mRNAseq, have been developed and applied for SNP discovery (Trick *et al*., 2009), linkage mapping and genome characterization (Bancroft *et al*., 2011), and transcript quantification (Higgins *et al*., 2012). Indeed, AT was first developed in *B. napus* with a very small genetic diversity panel, enabling the implication of orthologues of *HAG1* in the control of seed glucosinolate content (Harper *et al*., 2012).

**Figure 1:**
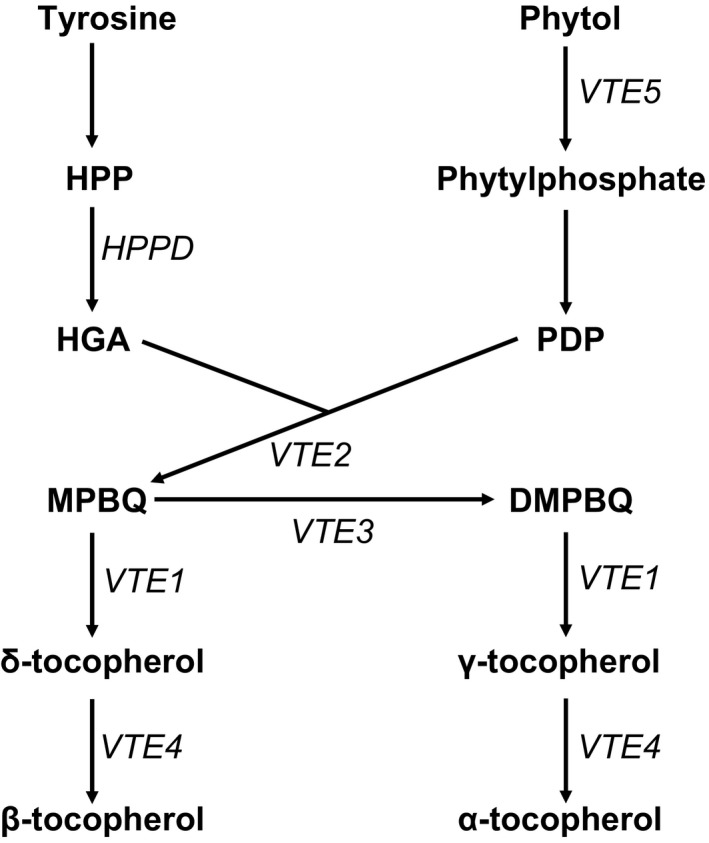
Simplified tocopherol biosynthesis pathway in plants. Abbreviations: DMPBQ, 2,3‐dimethyl‐5‐phytyl‐1,4‐benzoquinone; HGA, homogentisic acid; HPP,* p*‐hydroxyphenylpyruvate; *HPPD*, HPP dioxygenase; MPBQ, 2‐methyl‐6‐phytyl‐1,4‐benzoquinone; PDP, phytyl‐diphosphate; *VTE1*, tocopherol cyclase; *VTE2*, homogentisate phytyltransferase; *VTE3*, MPBQ methyltransferase; *VTE4*, γ‐tocopherol methyltransferase; *VTE5*, phytol kinase.

Vegetable oils are a major source of dietary vitamin E (Goffman and Becker, 2002). Vitamin E occurs in the form of tocopherols, which are lipid‐soluble antioxidants that accumulate in the chloroplast. Their function is to protect photosystem II from oxidative damage under the influence of free/released lipid peroxyl radicals and singlet oxygen (Quadrana *et al*., 2013), and in seeds they play a role in preventing the oxidation of polyunsaturated fatty acids (PUFAs). The four forms of tocopherol (α, β, γ and δ) vary in the number and position of methyl substituents attached to the chromanol ring (Munné‐Bosch and Alegre, 2002). The most abundant forms of vitamin E in rapeseed oil are γ‐ and α‐tocopherol, with a small proportion of δ‐tocopherol (Fritsche *et al*., 2012; Wang *et al*., 2012). Besides its nutritional value, α‐tocopherol is the most potent vitamin E, whereas the γ‐ and δ‐tocopherol forms are valued for their oil‐stabilizing properties (Munné‐Bosch and Alegre, 2002), which is particularly relevant for PUFA‐rich oils such as rapeseed. Tocopherol content and composition in rapeseed varies widely: values for total tocopherol content (TTC) have been reported to range between 166 and 687 mg kg^−1^, α‐tocopherol content ranges between 59 and 286 mg kg^−1^, and γ‐tocopherol content ranges between 107 and 280 mg kg^−1^. The ratio between α‐ and γ‐tocopherol has also been reported to range between 0.33 and 2.14 (Dolde *et al*., 1999; Goffman and Becker, 2002; Fritsche *et al*., 2012; Wang *et al*., 2012). Genes involved in the tocopherol biosynthetic pathway have been identified in *A. thaliana* and other model plants (Valentin *et al*., 2006; Endrigkeit *et al*., 2009; Li *et al*., 2012; Figure 1). Quantitative trait loci (QTL) affecting seed tocopherol content and composition have also been reported (Gilliland *et al*., 2006), but the control of biosynthesis is poorly understood.

The first AT panel reported for *B. napus* (Harper *et al*., 2012) comprised only 84 accessions and was smaller than is usually required for association studies (Spencer *et al*., 2009), meaning that it could be used successfully only for traits with a simple genetic basis. In this study, we report the establishment of a full AT platform for the crop species *B. napus*, based on a widely shared genetic diversity panel of 383 accessions, which can be used to address the genetic architecture of a broad range of traits. We validated the resource by using the new platform to analyse a trait that had been analysed previously using the original panel (erucic acid content of seed oil) and a new trait (the relative content of γ and α forms of tocopherol in seeds).

## Results

### The Renewable Industrial Products from Rapeseed (RIPR) genetic diversity panel

A diversity panel of 383 *B. napus* doubled haploid (DH) or inbred accessions was assembled, with the aim of covering the breadth of genetic variation available in the species. This panel included the breadth of crop types of *B. napus*, and comprised 362 inbred lines previously used by Bus *et al*. (2011) and Harper *et al*. (2012) plus 21 further accessions used by Thomas *et al*. (2016). The list of accessions is shown in Appendix S1. The panel is named RIPR after the research project ‘BBSRC Renewable Industrial Products from Rapeseed (RIPR) Programme’ that funded its development and genotyping.

### Functional genotypes

Functional genotypes were produced for the panel based on leaf RNA, with 100‐base read length mRNAseq data produced using the Illumina HiSeq 2000 platform. A total of 1.92 × 10^12^ bases of sequence data were produced. The sequence reads were mapped to the CDS gene model‐based Brassica AC pan‐transcriptome reference (He *et al*., 2015), which comprised 116 098 gene models, has an aggregate length of 118 657 829 bases and for which we provide an updated gene order based on a high‐density single‐nucleotide polymorphism (SNP) linkage map, as shown in Appendix S2. Sequence read mapping statistics are summarised in Appendix S1. Mean values of 50 165 125 reads were generated per accession, with 32 275 718 being mapped across 61 620 266 bases of the reference sequence, representing 52.1‐fold coverage of the 51.9% of the predicted transcriptome to which mRNAseq reads were mapped. SNPs were identified and gene expression quantified. Across the panel of 383 lines, 355 536 SNPs were scored, of which the majority (87.0%) were hemi‐SNPs, as found in previous *B. napus* studies (Trick *et al*., 2009). A total of 127 153 561 allele calls were made, with 9 017 727 (6.6%) missing values. Significant expression (>0.4 reads per kilobase per million mapped reads, RPKM) was detected for 53 889 CDS models (46.4% of all CDS models in the AC pan‐transcriptome reference), of which 25 834 belong to the A genome and 28 055 belong to the C genome. The functional genotypes are available from the York Oilseed Rape Knowledgebase (http://www.yorknowledgebase.info/).

### Genetic architecture of the population

**Figure 2:**
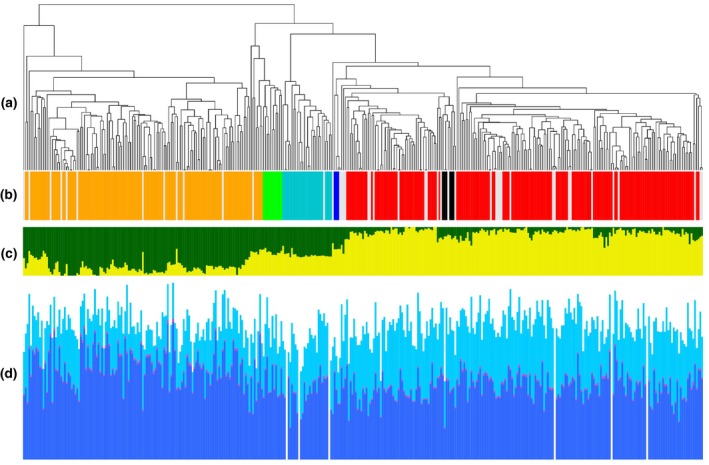
Population structure and trait variation across the Renewable Industrial Products from Rapeseed (RIPR) panel. (a) Relatedness of accessions in the panel based on 355 536 scored single‐nucleotide polymorphisms (SNPs). (b) Main crop types in the panel, colour‐coded: orange for spring oilseed rape; green for semi‐winter oilseed rape; light blue for swede; dark blue for kale; black for fodder; red for winter oilseed rape; and grey for crop type not assigned. (c) Population structure for highest likelihood *k* = 2. (d) Variation for seed content of α‐tocopherol (light blue), γ‐tocopherol (dark blue) and δ‐tocopherol (magenta).

The 355 536 SNP markers scored across the RIPR panel were used to analyse the relatedness of members of the panel. First, a distance matrix was generated and visualized by the dendrogram shown in Figure 2a. The assigned crop types (Appendix S1) show the expected clustering, as shown in Figure 2b. Next, the population structure of the panel was analysed using psiko (Popescu *et al*., 2014). The highest likelihood is a subpopulation *k *= 2, with mixture across the panel as illustrated in Figure 2c. Finally, LD was calculated across the genome, as summarised in Figure S1, producing a mean value of 0.031 for the population.

### Seed erucic acid analysis

Erucic acid is a 22‐carbon monounsaturated fatty acid. Its content in rapeseed oil is one of the key determinants of suitability for use as an edible or industrial oil. Detection of the known loci controlling the biosynthesis of erucic acid in seeds was used as a validation study for the first report of AT (Harper *et al*., 2012). We re‐analysed this trait to compare the performance of the original panel with the new RIPR panel. The fatty‐acid composition of seeds was determined for 376 lines of the RIPR diversity panel (summarised in Appendix S3). The erucic acid content of seeds varied between 0 and 51%, reflecting the range of crop types represented in the panel, which included modern Canola quality rapeseed varieties as well as crop types for which seed composition was not the subject of an active domesticated selection process (hence representative of ‘unimproved’ seed composition).

### Associative Transcriptomics of erucic acid content

**Figure 3:**
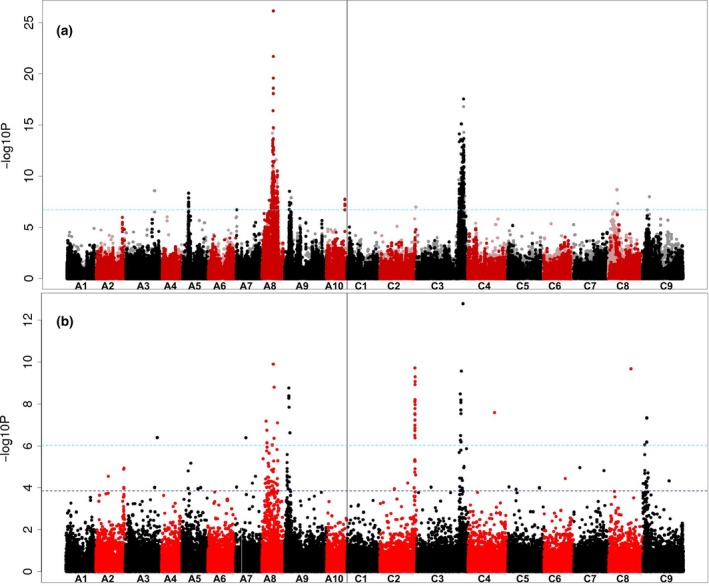
Association analysis. (a) Transcriptome single‐nucleotide polymorphism (SNP) markers with seed erucic acid content. The SNP markers are positioned on the *x*‐axis based on the genomic order of the gene models in which the polymorphism was scored, with the significance of the trait association, as –log10*P*, plotted on the *y*‐axis. A1–A10 and C1–C9 are the chromosomes of *Brassica napus*, shown in alternating black and red colours to permit boundaries to be distinguished. Hemi‐SNP markers (i.e. polymorphisms involving multiple bases called at the SNP position in one allele of the polymorphism) for which the genome of the polymorphism cannot be assigned are shown as light points, whereas simple SNP markers (i.e. polymorphisms between resolved bases) and hemi‐SNPs that have been directly linkage‐mapped, both of which can be assigned to a genome, are shown as dark points. The broken light‐blue horizontal line marks the Bonferroni‐corrected significance threshold of 0.05. (b) Transcript abundance with seed erucic acid content. The gene models are positioned on the *x*‐axis based on their genomic order, with the significance of the trait association, as –log10*P*, plotted on the *y*‐axis. The broken dark‐blue horizontal line marks the 5% false discovery rate.

The first stage of validation of the new AT platform for *B. napus* involved the analysis of seed erucic acid content, a trait for which the two main control loci are known and were confirmed previously by AT (Harper *et al*., 2012). The estimated narrow‐sense heritability (*h* ^2^) for the erucic acid trait was estimated from the SNP analysis as 0.794. A total of 318 genome‐assigned SNP markers above the Bonferroni‐corrected significance threshold of *P* = 0.05 (i.e. −log_10_ *P* value of 6.7) were detected across association signals on chromosomes A5, A8, A9, A10 and C3 (Appendix S4; Figure S3), as illustrated in Figure 3a. The main loci controlling erucic content (on chromosomes A8 and C3) provide association signals with a significance eight orders of magnitude greater: −log_10_ *P* > 16, compared with <8 in the previous study. The known control genes, orthologues of *FAE1* (AT4G34520), represented by gene models Cab035983.1 and Bo3g168810.1, are near the centres of these SNP association peaks, within six genes (approximately 42 kb) and nine genes (approximately 56 kb) from the closest significantly associated gene, respectively, according to the reference sequence (Appendix S4). In addition, SNP associations were found for a region of the genome, on chromosome A5, which were not previously detected. This indicates the position of a novel locus with minor effect on the trait. A candidate for the trait control gene in this region is Cab033920.1. This gene is an orthologue of AT2G34770.1, annotated as fatty acid hydroxylase 1, which has a potential role in very long chain fatty‐acid biosynthesis. An association signal was also detected for a relatively large region of chromosome A9, which we interpret as corresponding to a seed glucosinolate‐controlling locus, which was co‐selected in modern low erucic rapeseed cultivars to produce Canola quality seed.

In addition to association analysis using SNP markers, AT also reveals associations between gene expression markers (in the tissue of second true leaves used for the development of functional genotypes) and trait variation. In the case of seed erucic acid content, the main control genes (orthologues of *FAE1*) are transcriptionally inactive in the tissue (leaves) sampled for the production of the functional genotypes. We are still able to detect both SNP and gene expression marker (GEM) association peaks through markers in LD with *FAE1* on A8 and C3, however, as illustrated in Figure 3b. The lower resolution observed for the A8 peaks may reflect the influence of two strong bottlenecks during breeding selection (Hasan *et al*., 2008) for low glucosinolate content (controlling loci on chromosomes A2, A9, C2 and C9) and zero seed erucic acid content (controlling loci on chromosomes A8 and C3), or perhaps the presence of additional minor effect genes located on A8 that also contribute to the erucic trait. Indeed there are many potential candidate genes in the region that could have an effect, including an orthologue of FAD6 (AT4G30950), which could act to reduce the pool of oleic acid available for elongation to erucic acid. In addition, there is a signature of slightly inflated LD on the first half of A8, which may further contribute to reducing the resolution of association peaks in this region (Figure S1).

The clear signals in the transcript abundance‐based association analysis confirms the stability of differential gene expression across the panel, and its utility for the identification of association signals. Regions of the genome previously associated with seed glucosinolate content (selected alongside erucic content in Canola quality rapeseed) show particularly strong transcript abundance associations, which we interpret as consequences of the extensive structural variation in these regions of the genome (He *et al*., 2016). The new AT platform generates strong signals because of the large, diverse panel and superior number of markers assigned to homoeologues, properties lacking in the platform reported previously (Harper *et al*., 2012).

### Tocopherol phenotype analysis

We selected tocopherols in seeds as test traits of unknown genetic basis, quantifying α, γ and δ forms. Tocopherols were purified from seeds and quantified for 377 accessions of the RIPR panel. The results are summarised in Appendix S5 and Figure S2. Total tocopherol in seeds varied from 197 to 445 mg kg^−1^, with the main types being γ‐tocopherol (78–347 mg kg^−1^) and α‐tocopherol (51–229 mg kg^−1^), the relative proportions of which (measured as the γ/α‐tocopherol ratio) varied greatly, ranging from 0.485 to 5.00, with δ‐tocopherol representing a minor component (1.8–9.9 mg kg^−1^). Analysis of tocopherol characteristics by crop type showed that γ‐tocopherol content tended to be higher in spring crop types and α‐tocopherol content tended to be higher in winter crop types, as illustrated in Figure 2d.

Given that the purpose of tocopherols in seed oil is to protect against oxidation, we assessed the diversity panel for correlations of tocopherol traits with the proportions of the fatty acids found in seed oil that are most susceptible to oxidation, the PUFAs linoleic and linolenic. The content of these fatty acids had been determined alongside that of erucic acid (Appendix S3). A weak positive correlation between total tocopherol and PUFA content was, indeed, identified (*R* ^2^ = 0.13; *P *< 0.001).

### Associative Transcriptomics of tocopherol composition

**Figure 4:**
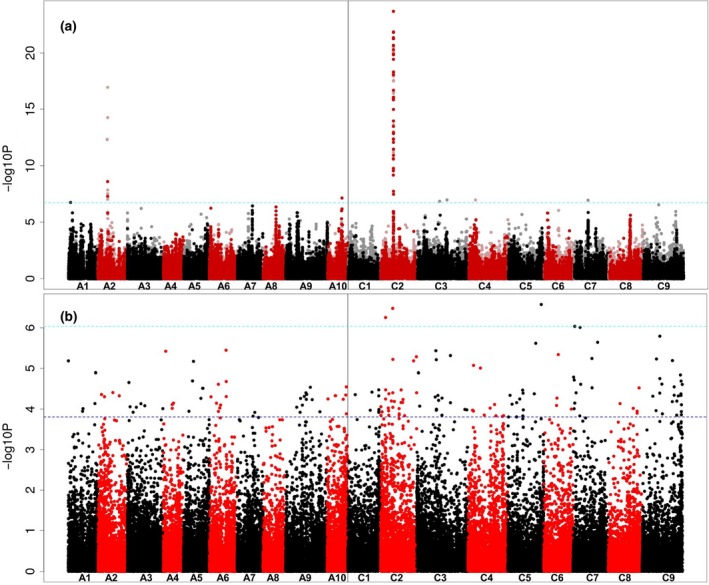
Association analysis. (a) Transcriptome single‐nucleotide polymorphism (SNP) association analysis for seed γ/α‐tocopherol ratio. The SNP markers are positioned on the *x*‐axis based on the genomic order of the gene models in which the polymorphism was scored, with the significance of the trait association, as –log10*P*, plotted on the *y*‐axis. A1–A10 and C1–C9 are the chromosomes of *Brassica napus*, shown in alternating black and red colours to permit boundaries to be distinguished. Hemi‐SNP markers (i.e. polymorphisms involving multiple bases called at the SNP position in one allele of the polymorphism) for which the genome of the polymorphism cannot be assigned are shown as light points, whereas simple SNP markers (i.e. polymorphisms between resolved bases) and hemi‐SNPs that have been directly linkage‐mapped, both of which can be assigned to a genome, are shown as dark points. The broken light‐blue horizontal line marks the Bonferroni‐corrected significance threshold of 0.05. (b) Association analysis of transcript abundance with seed γ/α‐tocopherol ratio. The gene models are positioned on the *x*‐axis based on their genomic order, with the significance of the trait association, as –log10*P*, plotted on the *y*‐axis. The broken dark‐blue horizontal line marks the 5% false discovery rate.

To undertake AT for tocopherol traits, we analysed the population for loci controlling the proportion of tocopherol occurring in the γ form rather than the α form by using the γ/α ratio as the trait. The SNP‐based association analysis, as illustrated in Figure 4a, revealed exceptionally strong associations with markers in a very small region of chromosome C2, along with weaker associations with a few markers in regions of chromosomes A2 and A10. Unlike seed erucic acid, tocopherol composition has not been selected for by *B. napus* breeders. We interpret the very sharp association signal as indicative of this lack of selection, and consider this to be consistent with LD across most of the genome. The association peak on chromosome C2 includes 33 genome‐assigned markers above the Bonferroni‐corrected significance threshold (alpha = 0.05; –log_10_ *P* value of 6.7; Appendix S6; Figure S3). These delineated a genomic region containing 39 genes, including an orthologue of *VTE4*, which encodes γ‐tocopherol methyl transferase (γ‐TMT), an enzyme that converts γ‐tocopherol into α‐tocopherol (Figure 1). A homoeologous region including a duplicate copy of the *VTE4* gene within the association peak on chromosome A2 was observed, whereas there was no obvious candidate gene in the region of chromosome A10 showing associations. Four transcript abundance‐based markers above the Bonferroni‐corrected significance threshold (–log_10_ *P* value of 6.03 for GEMs) were identified on chromosomes C2, C5 and C7 (Figure 4b). The identification of the gene *VTE4* as the most highly associated GEM on chromosome C2 demonstrated the ability for AT to efficiently provide candidate genes associated with traits of interest.

**Figure 5:**
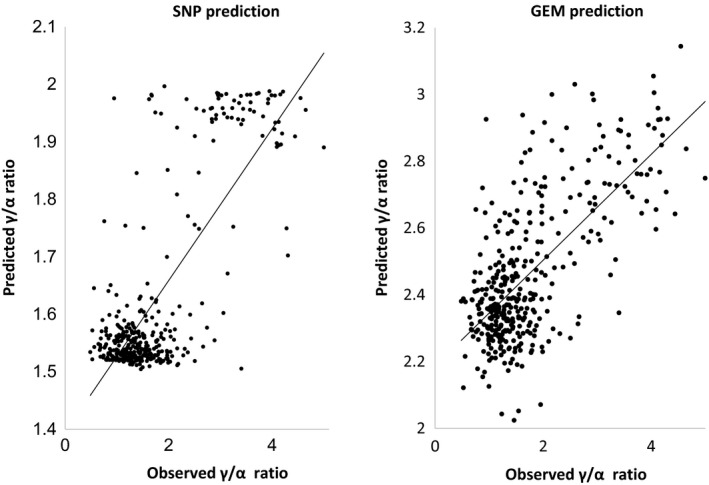
Test of the predictive ability of single‐nucleotide polymorphisms (SNPs) and gene expression markers (GEMs) associated with γ/α‐tocopherol ratio by ‘take‐one‐out’ permutation. The allelic effects of each of 36 SNP markers associated with the γ/α‐tocopherol ratio was used to predict the γ/α‐tocopherol ratio for the missing accessions. For GEM data, reads per kilobase per million (RPKM) values for each of four GEMs were fitted to the regression line to predict the γ/α‐tocopherol ratio. The strong correlation between predicted and observed γ/α‐tocopherol ratio values (*R* ^2^ = 0.59, *P *< 0.001 for SNPs; *R* ^2^ = 0.47, *P *< 0.001 for GEMs) demonstrates excellent predictive ability.

To investigate whether the top selected markers are predictive for the γ/α ratio, we performed a set of ‘take‐one‐out’ permutations for the SNP and GEM markers identified from association analysis of 377 accessions adapted from Harper *et al*. (2016). Markers above the Bonferroni line (Appendixes S6 and Appendix S7) were selected for each round of permutations. For SNP data, the allelic effects of each of these markers was used to predict trait values for the missing accessions based on their scored genotypes. For GEM data, RPKM values were fitted to the regression line to predict trait values. The predicted trait values against the observed traits are illustrated as scatter plots in Figure 5, and confirmed their excellent predictive ability (*R* ^2^ = 0.59 for SNPs and *R* ^2^ = 0.47 for GEMs between predicted and observed values; *P *< 0.001), which reflect the estimated narrow‐sense heritability (*h* ^2^) of 0.452 for the γ/α ratio. These SNPs and GEMs can therefore be used as promising markers in marker‐assisted breeding.

**Figure 6:**
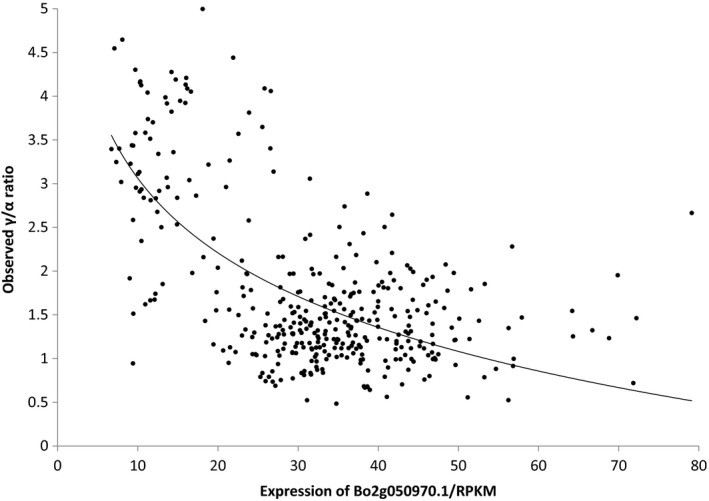
Relationship between the expression of *Bo2g050970.1* in leaves and the γ/α‐tocopherol ratio in seeds. The ratio of γ/α‐tocopherol measured in seeds was regressed against the transcript abundance in leaves of the VTE4 orthologue *Bo2g050970.1* (*R* ^2^ = 0.26; *P *< 0.001), measured as reads per kilobase per million aligned reads (RPKM).

In order to confirm the role of the *VTE4* orthologue in the associated region of C2 (Bo2g050970.1), we used the transcript quantification data that were obtained alongside the transcriptome SNP data as part of the functional genotypes. As illustrated in Figure 6, these show that the expression level of Bo2g050970.1 in the tissue sampled to produce the functional genotypes (leaves) is negatively correlated with the γ/α ratio (*R* ^2^ = 0.41, *P *< 0.001). This is consistent with the predicted γ‐TMT activity of the gene encoded by Bo2g050970.1 (i.e. lower expression leading to less conversion of γ‐tocopherol to α‐tocopherol). There had been no significant associations between SNPs within Bo2g050970.1 and the γ/α ratio, consistent with the basis of the allelic variation being variation in gene expression rather than variation in gene sequence.

## Discussion

Association studies are becoming increasingly widely used in crops for identifying molecular markers linked to trait‐controlling loci (Rafalski, 2010); however, polyploid crops present additional difficulties that must be overcome, including the intrinsic genome complexity and increased genome structural instability, such as the copy‐number variations (CNVs) that affect gene families (Zhang *et al*., 2013; Renny‐Byfield and Wendel, 2014). Such difficulties occur in *B. napus*, as was recently shown by Chalhoub *et al*. (2014) and He *et al*. (2016). Association studies have to meet many demands to maximize the probability of identifying marker–trait associations. In addition to good experimental design, along with access to all the necessary equipment and available funds, there is also the need to choose a permanent and sufficiently large set of diverse and preferably homozygous individuals, the larger size and higher genetic diversity of which providing sufficient power for association analysis (Spencer *et al*., 2009; Huang and Han, 2014). Once assembled, association panels need to be genotyped with molecular markers to a sufficiently high density to identify polymorphisms in linkage disequilibrium with trait‐controlling loci. The development of suitable association panels is challenging for individual research groups, providing a driver for the development of community resources.

In this study, we introduce a new genetically diverse AT panel of 383 rapeseed accessions, together with a mapping platform that comprises complete genotype information for this panel, which may be used for a broad range of association studies suitable for re‐phenotyping any trait, without the need of additional genotyping. This panel, being made available with all transcriptomic data, offers a large range of potential applications: identifying causative genes, uncovering unknown pathways, identifying regulatory genes or transcription factors, and screening of available germplasm for allelic variants and to support the development of molecular markers for marker‐assisted breeding. Our resource provides 355 536 SNP markers, equivalent to one SNP every 0.33 kb across our *B. napus* AC pan‐transcriptome reference. The SNP density is much higher than the density of the commercially available 60K Brassica Infinium^®^ SNP array, which only provided 26 841 or 21 117 SNPs for recent *B. napus* GWAS studies (Li *et al*., 2014; Xu *et al*., 2016). Although the number of SNPs can even be greater when using whole‐genome resequencing, as shown by Huang *et al*. (2013), the advantage of transcriptome resequencing using mRNAseq is the availability of transcript abundance data: in our case for 46% of the genes present in the AC pan‐transcriptome reference sequence. In this study, we demonstrate a significant step‐change in resolution from our original AT platform based on a panel of 84 accessions, as reported in Harper *et al*. (2012). The unigene‐based transcriptome reference sequence used by that platform had relatively poor capability to resolve homoeologous loci, because of its construction based on a Brassica‐wide transcriptome assembly and subsequent ‘curing’ to more closely match the progenitor genomes. In the absence of the ability to map sequence reads unambiguously to the correct homoeologue, most SNPs appear, because of cross‐mapping, as ‘hemi‐SNPs’, i.e. where one allele comprises a mixture of two bases (Trick *et al*., 2009). In the original platform only a small proportion of markers could be assigned with high confidence to a genome, the majority being assigned to both homoeologous positions. The new platform is based mainly on gene models originating from the genome sequences of the progenitor species, and permits more discriminating read‐mapping, resulting in a greater proportion of ‘simple SNPs’ (i.e. where the polymorphism is between resolved single bases only) that can be assigned with confidence to a genome. Where there are association peaks comprising pale points in homoeologous positions to the associations identified, such as those observed in the regions of A2 depicted in Figure 4a, these can be disregarded as homoeologous ‘shadows’ of the regions genuinely containing causative variation. The discovery of SNPs for particular genes from juvenile leaves can be limited by their transcription in different phenological stages or tissues, but candidate loci/genes associated with traits manifesting in different times or places can be identified, as demonstrated here in the case of *FAE1* and in previous AT studies (Lu *et al*., 2014; Wood *et al*., 2017). This is possible because of the presence of variation in genes in LD with the causative gene, resulting in an associated region including the control gene. In addition, the new platform provides much greater resolution of the contributions to the transcriptome of pairs of homoeologous genes. This permitted the efficient detection of association peaks based solely on transcript abundance variation, as illustrated in Figure 3. Moreover, the current platform also allows a deeper insight into the structural changes and functional interactions between *B. napus* AC genomes. Information about respective homologous genes, including their copy number, sequence variation and transcript prevalence provides important information in polyploid research.

In addition to extending previous association studies of the control of seed erucic acid content, a trait selected recently by rapeseed breeders, we applied the platform to a trait not previously selected by breeders or studied extensively: the control of tocopherol (vitamin E) forms accumulated in seeds. We analysed seed tocopherols in 377 rapeseed accessions for their type and content. The profiles presented here showed a high degree of variability for the γ/α‐tocopherol ratio (Coefficient of Variance = 53%), displaying distinct patterns for different crop types, that allowed us to identify gene *Bo2g050970.1* (an orthologue of the Arabidopsis gene *VTE4*) on chromosome C2 as a candidate gene, based on inference of gene function from studies of its orthologue in *A. thaliana*. Although there was no evidence of the presence of any specific allelic form of the *VTE4* orthologue associated with γ/α‐tocopherol ratio, this gene has been easily identifiable by the presence of SNPs in surrounding genes. This set of tightly linked markers exhibited excellent predictive ability (Figure 5), which we attribute to the broad (species‐wide) range of genetic variation represented by the RIPR diversity panel, overcoming the lack of predictive capability that can be encountered when applying markers to test material (Bush and Moore, 2012). The association that we observed between transcript abundance of *Bo2g050970.1* in leaves and the γ/α‐tocopherol ratio in seeds is consistent with our understanding that tocopherols are synthesized and localized in plastids and accumulate in all tissues, with generally the highest content in seeds (Sattler *et al*., 2004). In Arabidopsis, γ‐TMT (*VTE4,* AT1G64970) is known to use δ‐ and γ‐tocopherols as substrates to produce β‐ and α‐tocopherols, respectively (Shintani and DellaPenna, 1998), and the effect of the *VTE4* gene from *B. napus* on α‐tocopherol content has also been proven by overexpression in *Glycine max* (soya bean) and Arabidopsis (Endrigkeit *et al*., 2009; Chen *et al*., 2012).

By assembling and developing functional genotypes (i.e. comprising both gene sequence variation and gene expression variation) for a diversity panel representing species‐wide genetic diversity, we have established a resource for the whole rapeseed research community to use. Furthermore, the success of the approach of Associative Transcriptomics for the identification not only of linked markers but of candidates for causative genes serves as an exemplar for plant and crop science more broadly.

## Experimental procedures

### Growth of the genetic diversity panel

The panel of 383 *B. napus* accessions is available from the John Innes Centre ( https://www.jic.ac.uk). It was planted in a randomized block design of five biological replicates under controlled conditions of two polytunnels at the University of Nottingham, as described by Thomas *et al*. (2016). The accessions comprise inbred derivatives of both recent and historic varieties and some research lines. Plants were bagged before flowering to prevent cross‐pollination. Seeds were collected from individual plants at maturity. Seeds from 377 and 376 accessions were used for the tocopherol and erucic acid measurement, respectively. Based on descriptors originally received with the material and analysis of relatedness, they were attributed to one of seven different groups, namely spring oilseed rape (123), semi‐winter oilseed rape (11), swede (27), kale (3), fodder (6), winter oilseed rape (169) or crop type not assigned (44), as listed in Appendix S1.

### Measurement of fatty‐acid content and composition

For the analysis of fatty acid methyl esters (FAMEs), 30 mg of seeds were homogenized in a glass vial with 5 mL of heptane. To the homogenate, 500 μL of 2 M potassium hydroxide was added, left for 1 h and then neutralized with sodium hydrogen sulphate monohydrate. The upper phase was transferred into crimp‐cap Chromacol 0.8‐ml vials ( https://www.thermofisher.com) for analysis using a DANI Master GC fitted with an SGE‐BPX70 double column ( https://dani-instruments.com).

### Measurement of tocopherol content and composition

The α‐, γ‐ and δ‐tocopherol (the sum of which formed total tocopherol, TTC) were extracted from a homogenous mixture of 80 mg rapeseed seeds and analysed by normal‐phase HPLC, as described previously (Fritsche *et al*., 2012). Modified mobile phase A was heptane (Rathburn Chemicals Co., http://rathburn.co.uk), phase B was heptane:dioxane (90:10, v/v; Sigma‐Aldrich, https://www.sigmaaldrich.com). The internal standard, α‐tocopherol acetate (Sigma‐Aldrich), was added to each sample at a concentration of 25.4 μM (12 μg mL^−1^).

### SNP identification and transcript quantification for RNA‐seq data

The growth conditions, sampling of plant material, RNA extraction and transcriptome sequencing was carried out as described by He *et al*. (2016). The RNA‐seq data from each accession line were mapped onto recently developed ordered Brassica A and C pan‐transcriptomes (He *et al*., 2015) as reference sequences (maq 0.7.1; Li *et al*., 2008). SNPs were called by the meta‐analysis of alignments as described in Bancroft *et al*. (2011) of mRNAseq reads obtained from each of the *B. napus* accessions. SNP positions were excluded if they did not have a read depth in excess of 10, a base call quality above Q20, missing data below 0.25, and three alleles or fewer. An additional noise threshold was employed to reduce the effect of sequencing errors, whereby ambiguous bases were only allowed to be called if both bases were present at a frequency of 0.2 or above. This resulted in a set of 355 536 SNPs, of which 256 397 had the second most frequent allele in the population, so called here as a minor allele frequency (MAF) > 0.01. The markers were also classified as those that can be assigned with confidence to the genomic position of the CDS model in which they are scored (simple SNPs and hemi‐SNPs genetically mapped into the appropriate genome using the Tapidor Ningyou 7 Doubled Haploid (TNDH) mapping population), and those that cannot, as the polymorphism may be in either homoeologue of the CDS model in which they are scored (hemi‐SNPs not genetically mapped into the appropriate genome using the TNDH mapping population). Transcript abundance was quantified and normalized as reads per kb per million aligned reads (RPKM) for each sample for 116 098 CDS models of the pan‐transcriptome reference. Significant expression (> 0.4 RPKM) was detected for 53 889 CDS models.

### Clustering based on SNP genotypes

Clustering and dendrogram visualization on SNP data was performed by an r script developed in‐house. r package ‘phangorn’ was used for generating a distance matrix with the JC69 model (Schliep, 2011).

### Assessment of linkage disequilibrium

Pairwise LD was calculated and heat maps were produced for each individual chromosome, and these values were then used to calculate the mean LD across the genome. SNPs were removed from the analysis if they were not confirmed by TNDH population (Qiu *et al*., 2006) that assigned to the A or C genome, and if their minor allele frequency was below 0.01. A single SNP was selected at random from each CDS model to reduce the effect of many linked SNPs in the same gene. Pairwise *R* ^2^ LD matrices and heat maps were calculated for each chromosome using the r package ldheatmap 0.99‐2 (Shin *et al*., 2006).

### Associative Transcriptomic analysis

Association analysis for SNPs and GEMs was performed using r, as previously described (Harper *et al*., 2012; Sollars *et al*., 2017), with modifications. In order to deal with the greatly increased sizes of the data sets, psiko (Popescu *et al*., 2014) was used for Q‐matrix generation and the gapit r package was used with a mixed linear model (Lipka *et al*., 2012) for GWAS analysis. For Manhattan plots of SNP associations, SNP markers were filtered to include only those with minor allele frequencies of > 0.01: markers that could be assigned with confidence to the genomic position of the CDS model are rendered as dark points and markers that could not be assigned with confidence were rendered as pale points. For GEM association, CDS models were filtered prior to regression to include only those with mean expression across the panel of >0.4 RPKM. The association between gene expression and traits was calculated by fixed‐effect linear model in r, with RPKM values and the Q matrix inferred by psiko as the explanatory variables, and with trait score as the response variable. *R* ^2^ regression coefficients, constants and significance values were outputted for each regression. Genomic control (Devlin and Roeder, 1999) was applied to the GEM analysis to correct for spurious associations, with *P*‐value adjustment applied when the genomic inflation factor (λ) was observed to be greater than 1.

### Validation of marker association by trait prediction

The predictive power of the best GEMs and SNPs were assessed using a ‘take‐one‐out’ approach (Harper *et al*., 2016) whereby each accession is removed from the SNP or GEM analysis in turn. An in‐house r script was performed with adaptation from Harper *et al*. (2016), with a modification of incorporating all SNPs and GEMs above Bonferroni lines. When permutations finish, an *R* ^2^ value is calculated from predicted trait values regressed against the observed trait values, which indicates the predictive power of the top selected GEMs and SNPs.

## Accession numbers

Sequence data from this article can be found in the SRA data library under accession number PRJNA309367.

## Conflicts of Interest

The authors declare no conflicts of interest.

## Supporting Information

Supporting data are available. The largest data sets, representing the functional genotypes of the RIPR panel, are accessible via a data distribution website: http://www.yorknowledgebase.info/.The smaller data sets are hosted as supporting information online.

## Supporting information


**Figure S1.** Genome‐wide linkage disequilibrium analysis for the RIPR diversity panel.

**Figure S2.** Histograms of seed tocopherol composition of the RIPR diversity panel in different crop types.

**Figure S3.** Quantile–quantile plots from GEM and SNP association analysis for erucic acid and γ/α‐tocopherol ratio.

**Appendix S1.** List of cultivars, crop type classifications and Illumina read mapping statistics.

**Appendix S2.** Ordered list of CDS gene model‐based Brassica AC pan‐transcriptome.

**Appendix S3.** Seed fatty‐acid composition of the RIPR diversity panel.

**Appendix S4.** Markers and genomic regions showing association with variation for erucic acid content.

**Appendix S5.** Seed tocopherol composition of the RIPR diversity panel.

**Appendix S6.** Markers and genomic regions showing association with variation for γ/α‐tocopherol ratio.

**Appendix S7.** Gene expression markers showing association with variation for γ/α‐tocopherol ratio.
