# Supplementary material for: Validation of an updated Associative Transcriptomics platform for the polyploid crop species Brassica napus by dissection of the genetic architecture of erucic acid and tocopherol isoform variation in seeds
Source: Plant J. 2017 Dec 2;93(1):181–92. doi: 10.1111/tpj.13767 (PMC5767744; doi:10.1111/tpj.13767)

Pairwise LD in r^2 with 1178 SNPs in A01 cds range 5_to_5594 out of 5594

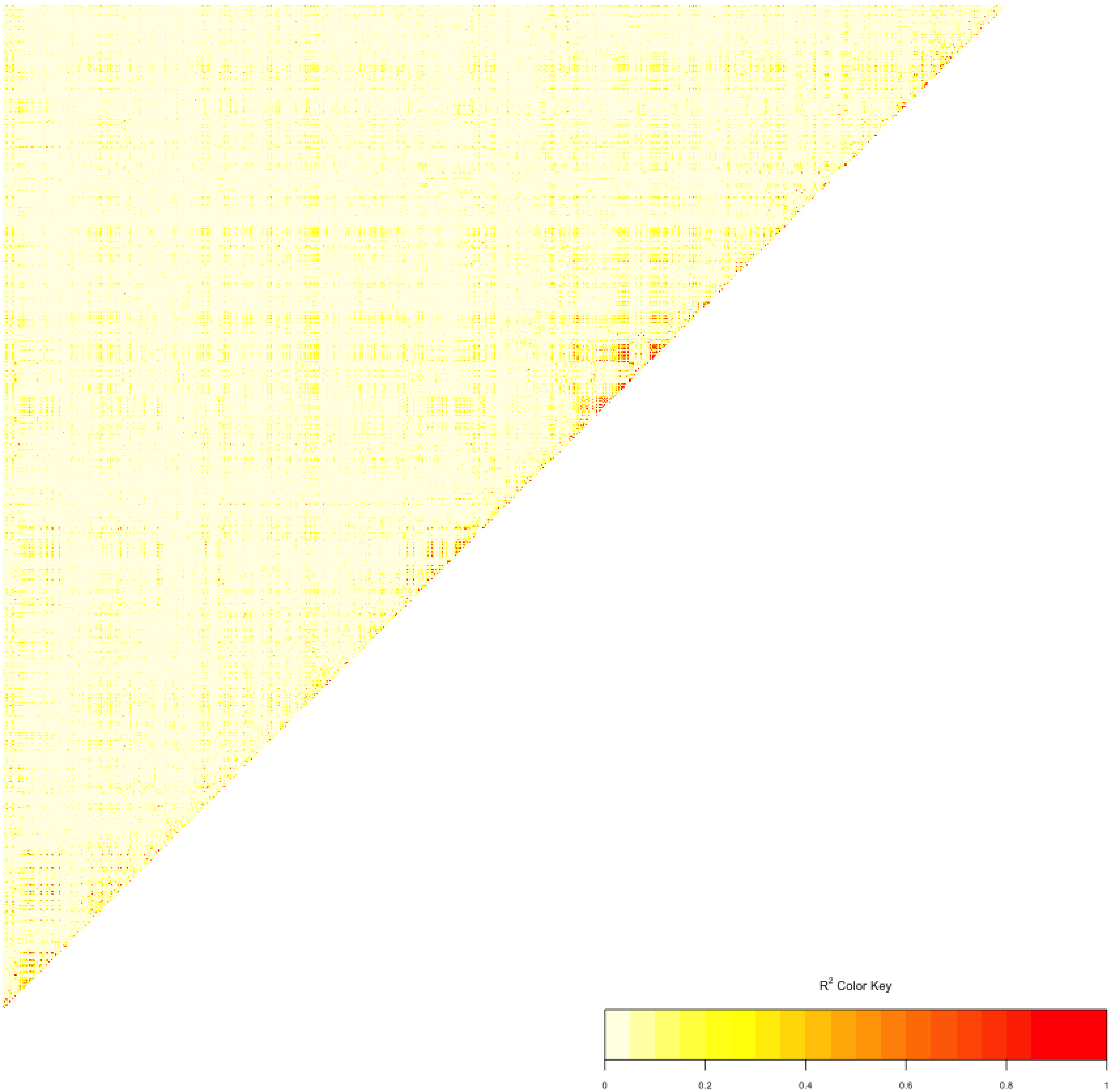

Pairwise LD in r^2 with 1085 SNPs in A02 cds range 6_to_5534 out of 5536

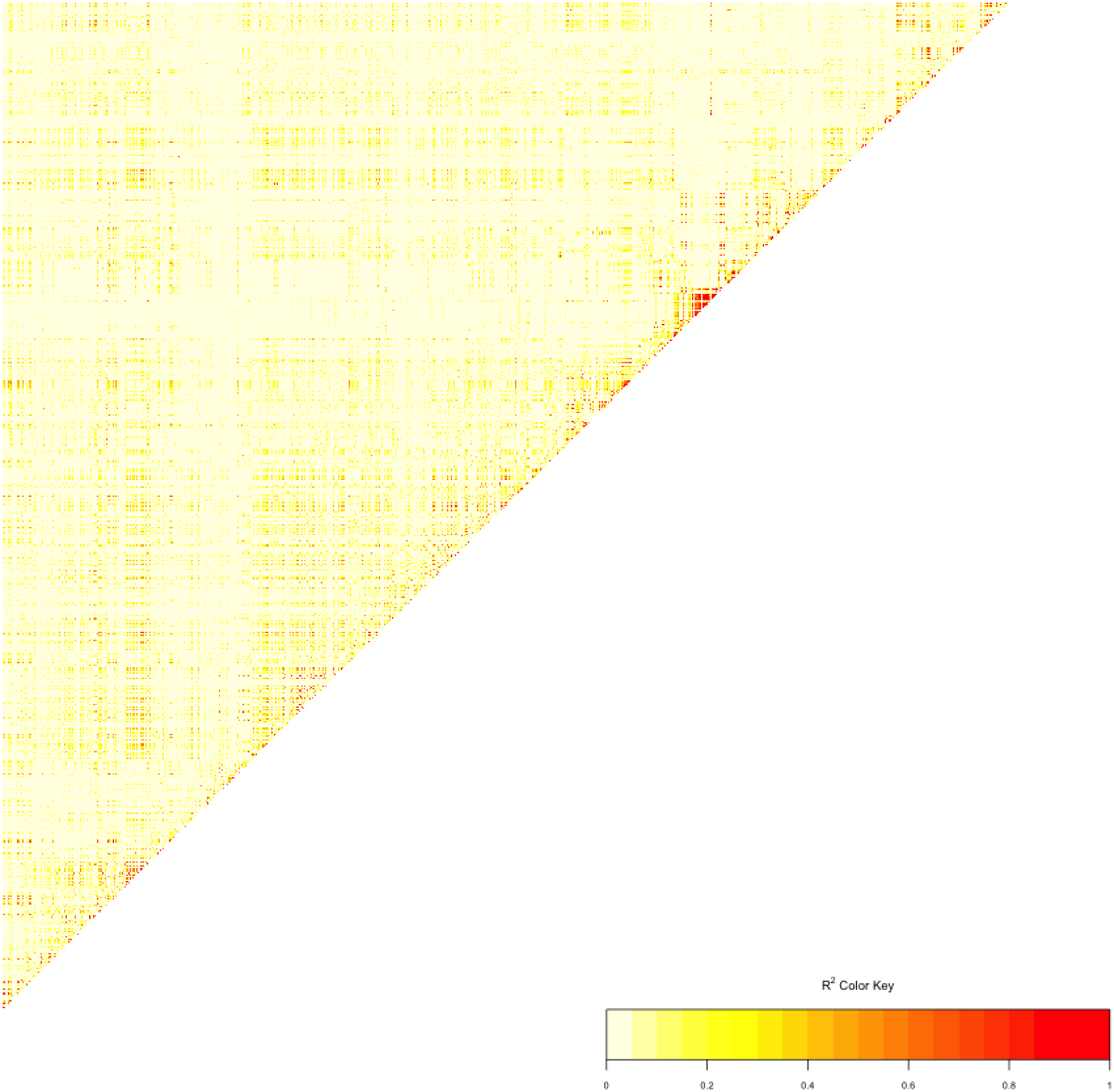

Pairwise LD in r^2 with 1759 SNPs in A03 cds range 5_to_6773 out of 6839

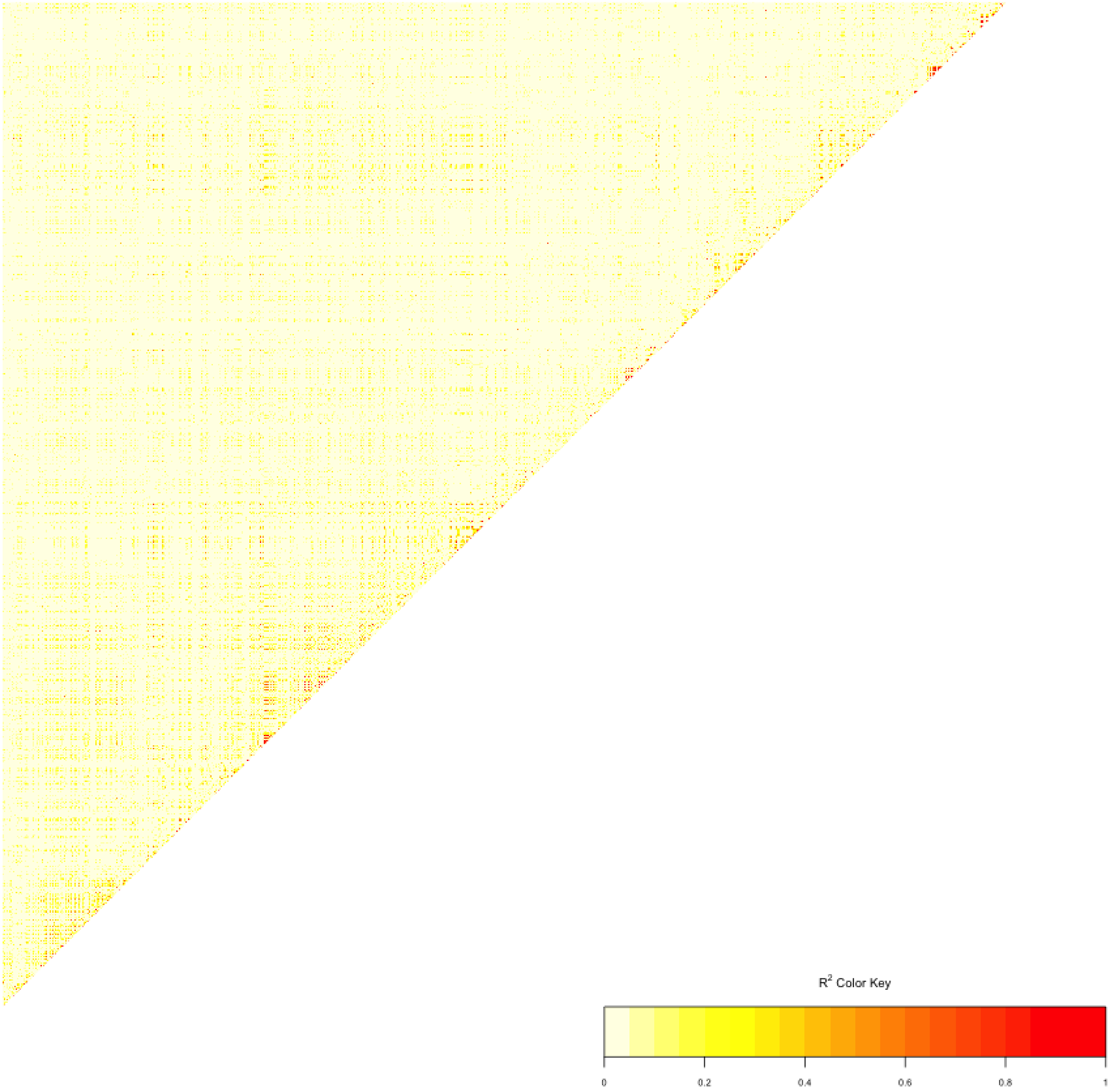

Pairwise LD in r^2 with 847 SNPs in A04 cds range 3_to_3805 out of 3810

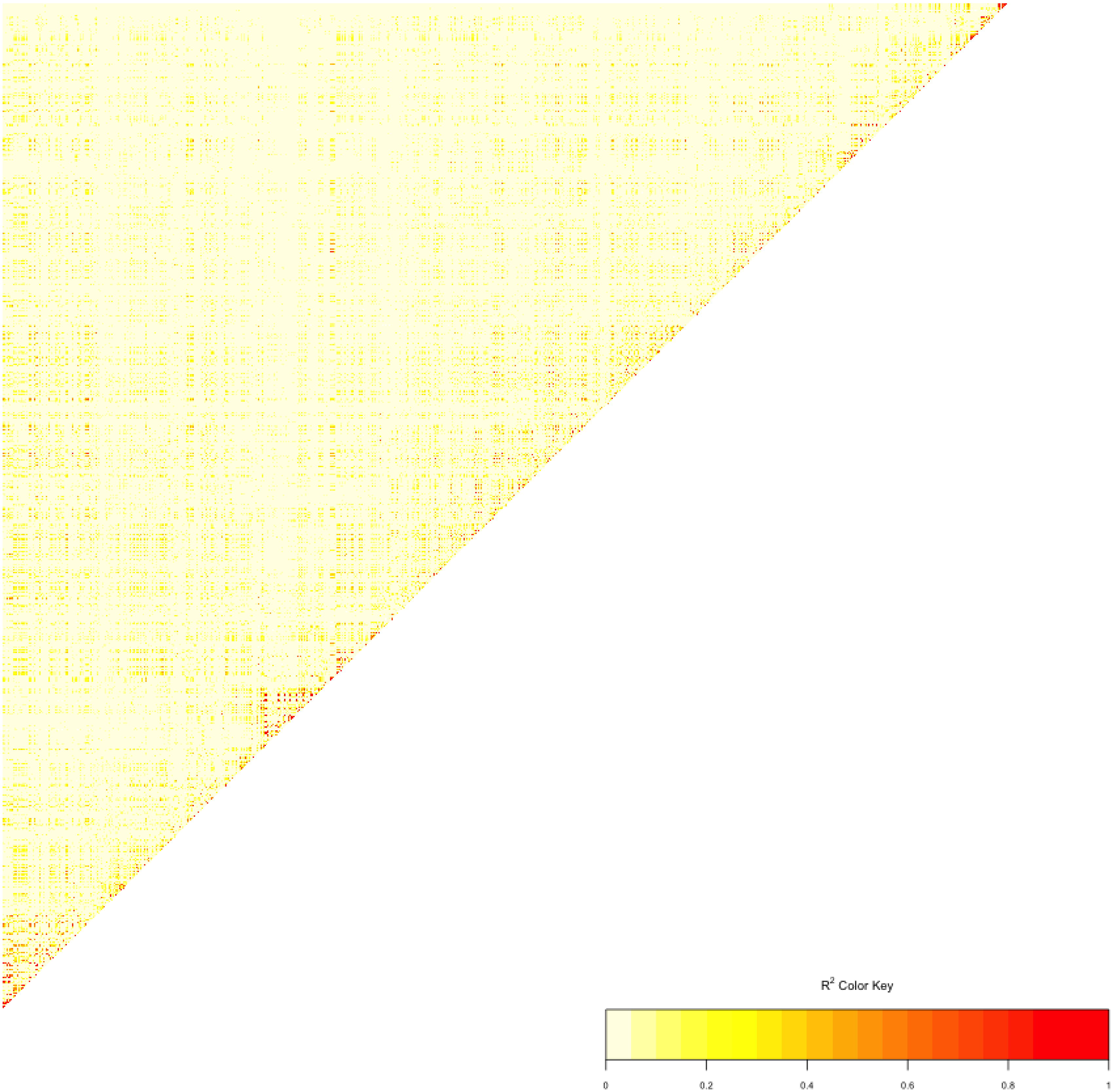

Pairwise LD in r^2 with 1162 SNPs in A05 cds range 9_to_4856 out of 4857

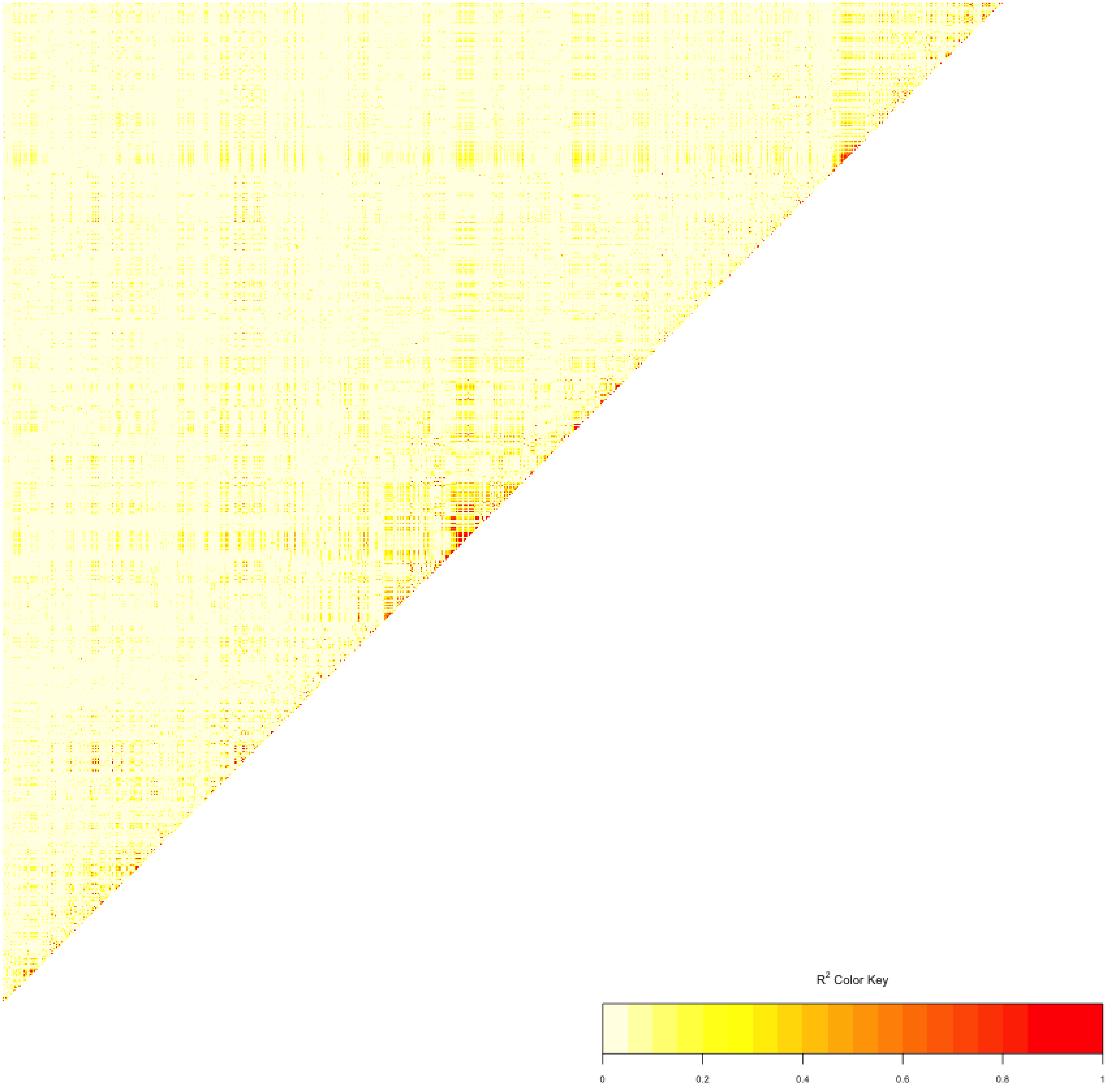

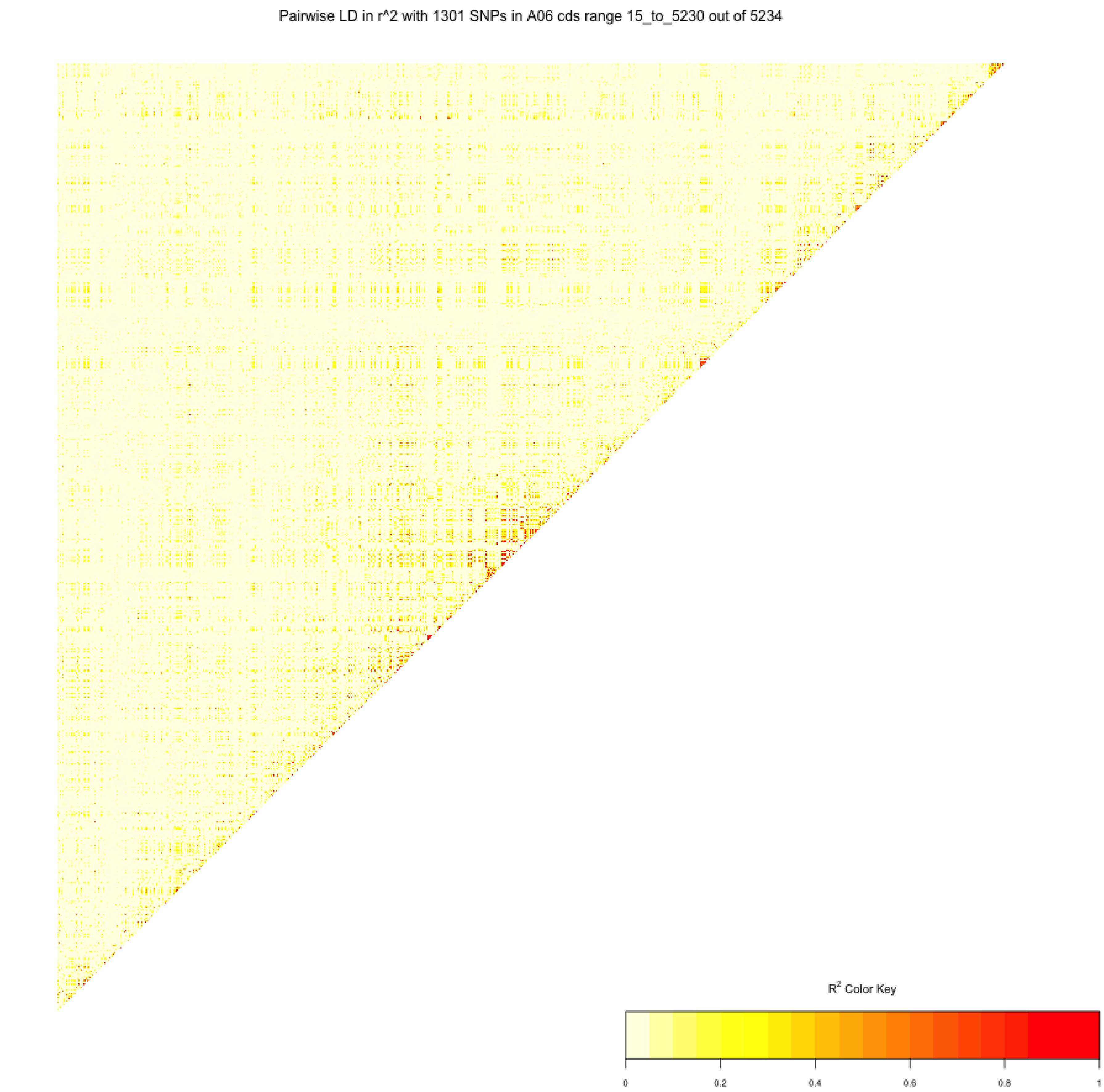
Pairwise LD in r^2 with 1301 SNPs in A06 cds range 15_to_5230 out of 5234
R^2 Color Key
0
0.2
0.4
0.6
0.8
1

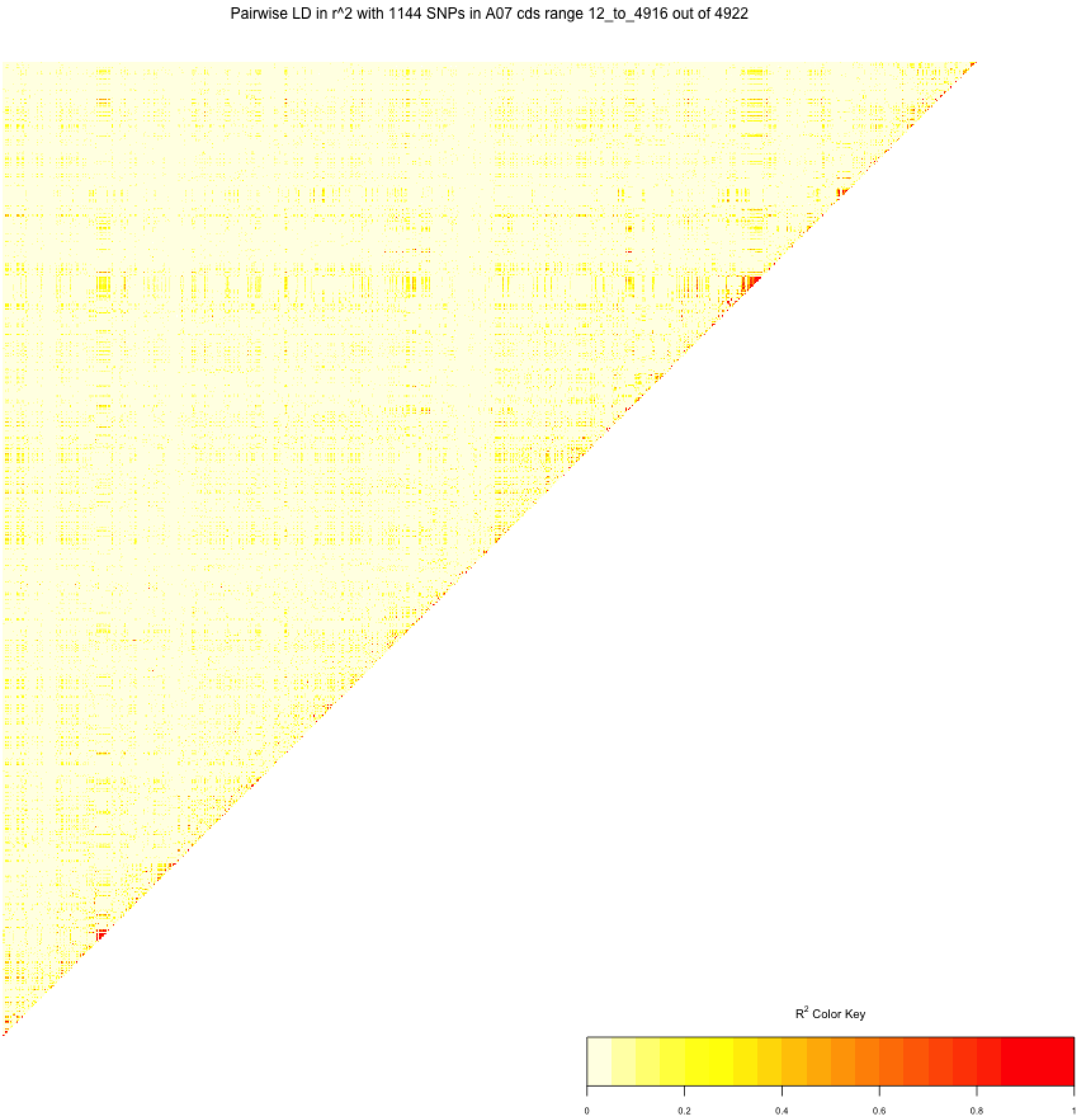
Pairwise LD in r^2 with 1144 SNPs in A07 cds range 12_to_4916 out of 4922
R2 Color Key
0
0.2
0.4
0.6
0.8
1

Pairwise LD in r^2 with 905 SNPs in A08 cds range 3_to_4265 out of 4268

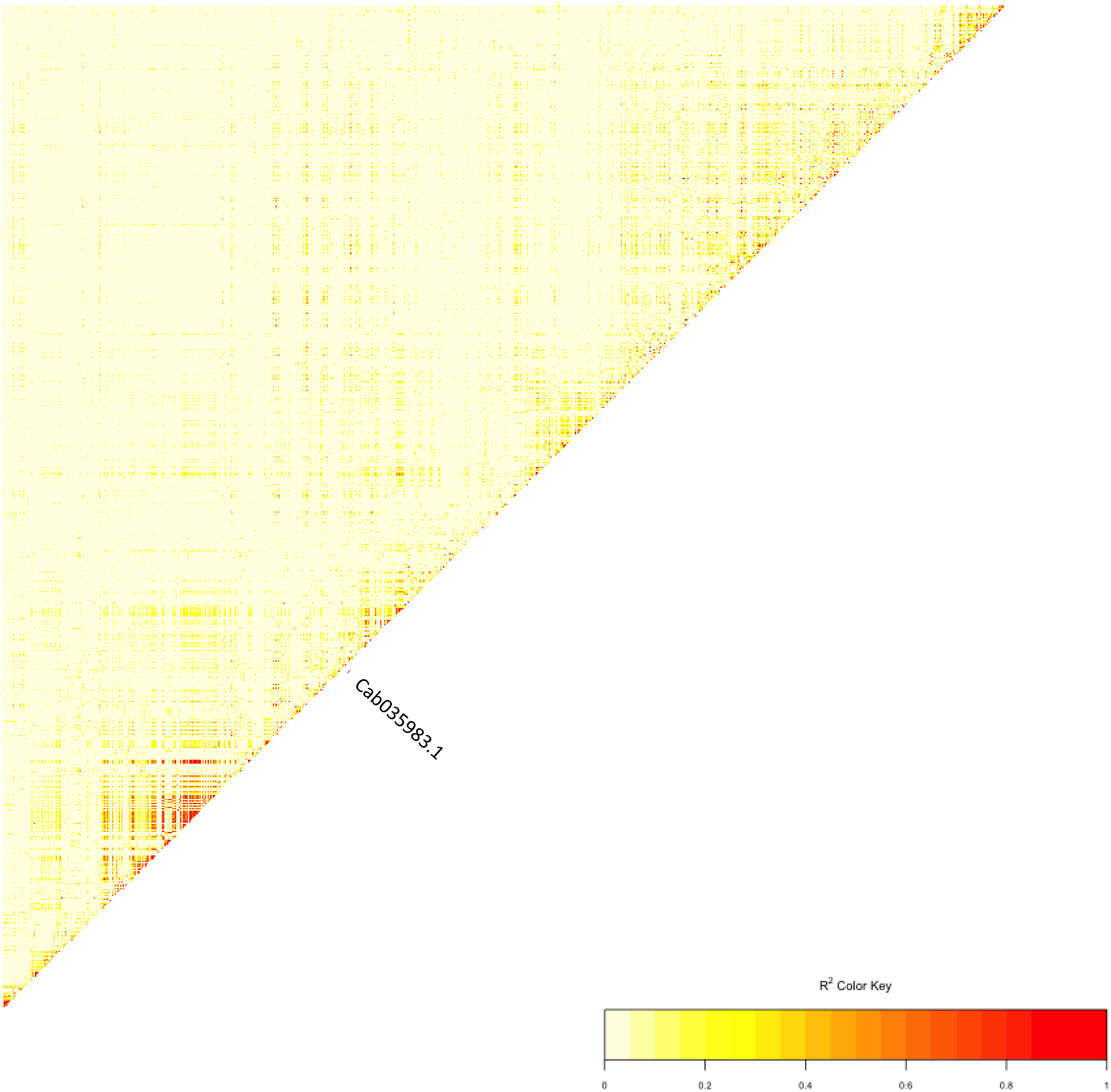

Pairwise LD in r^2 with 1763 SNPs in A09 cds range 2_to_7899 out of 7899

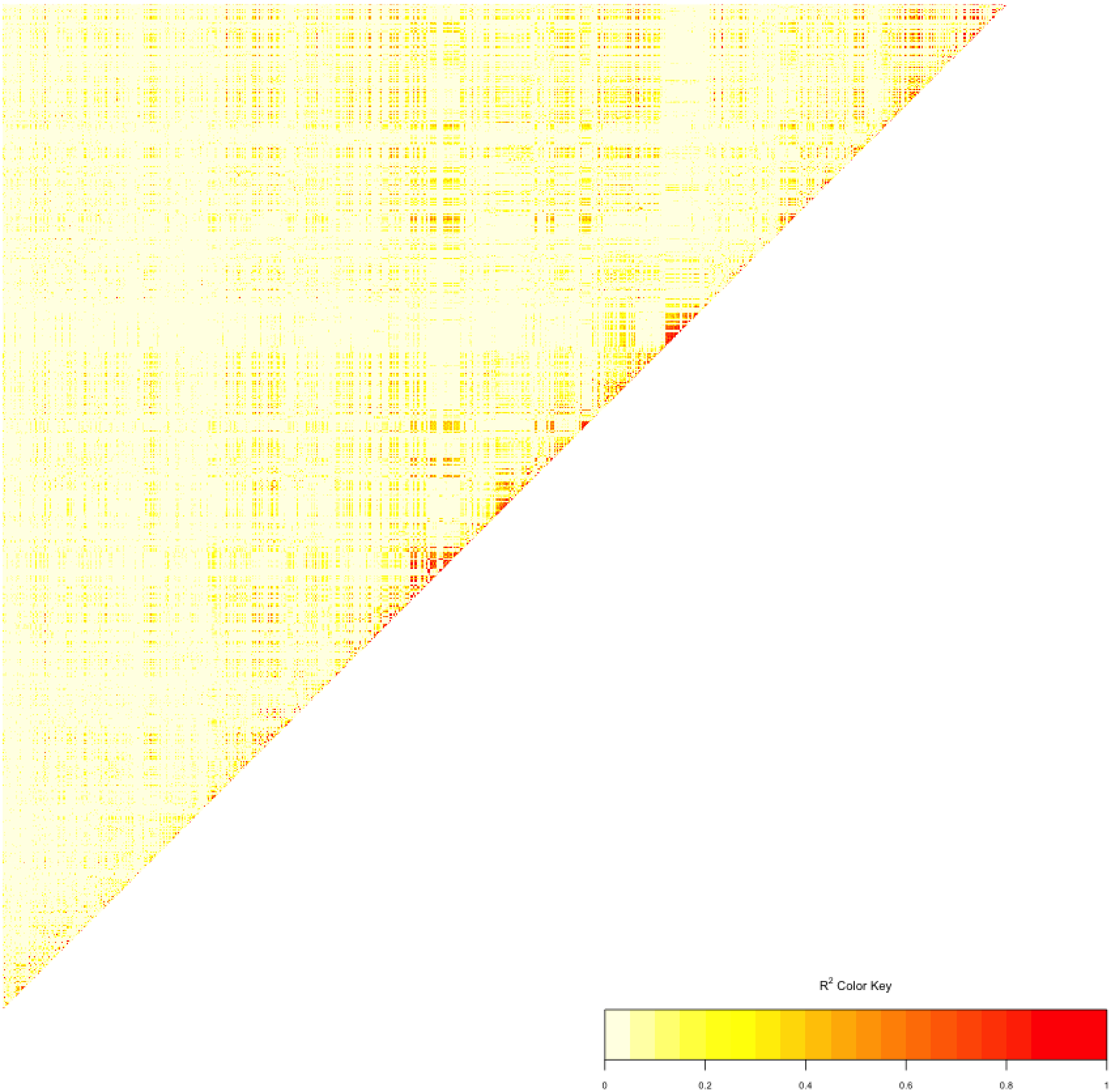

Pairwise LD in r^2 with 920 SNPs in A10 cds range 3_to_3819 out of 3831

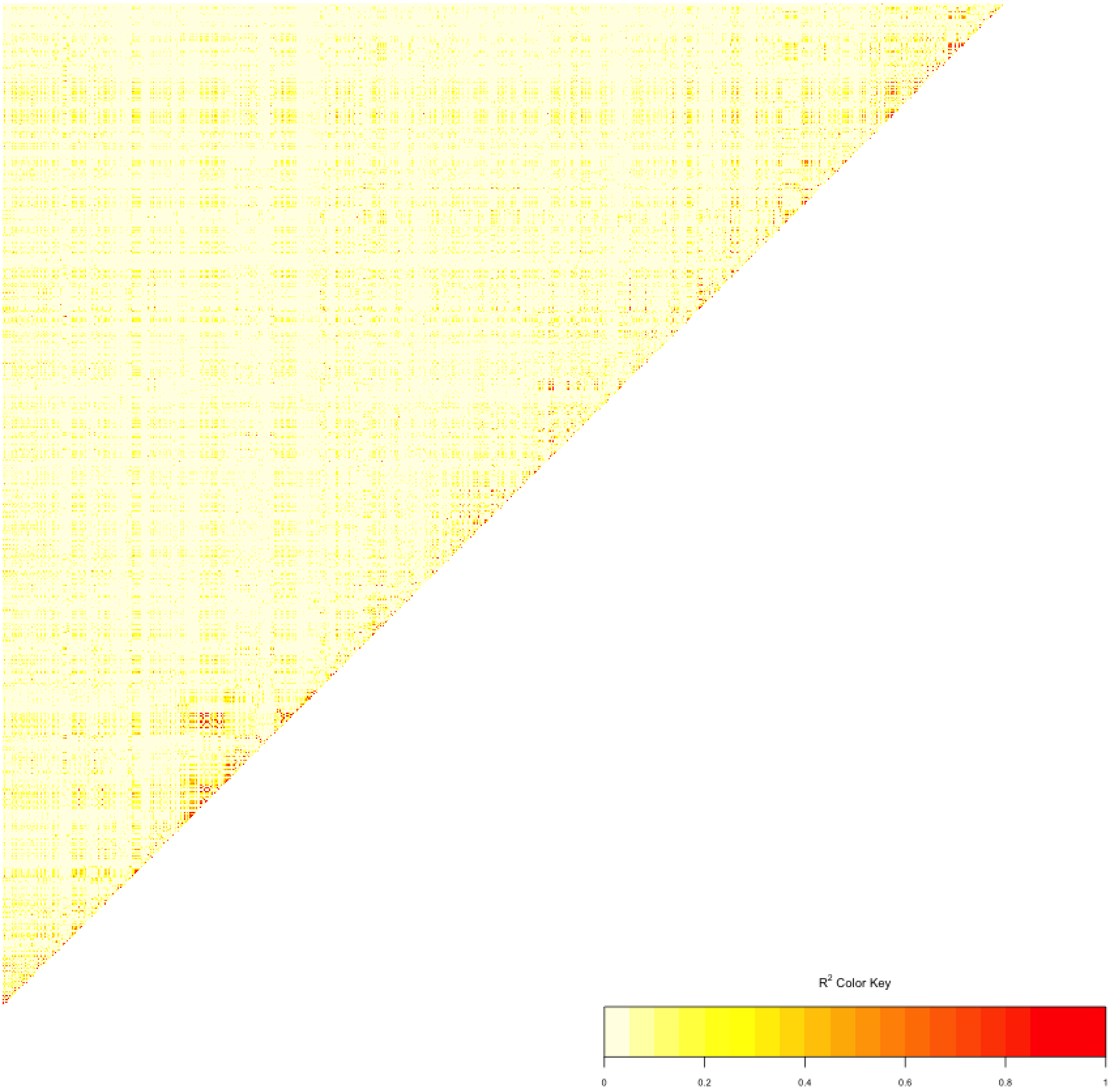

Pairwise LD in r^2 with 1079 SNPs in C01 cds range 5_to_6207 out of 6211

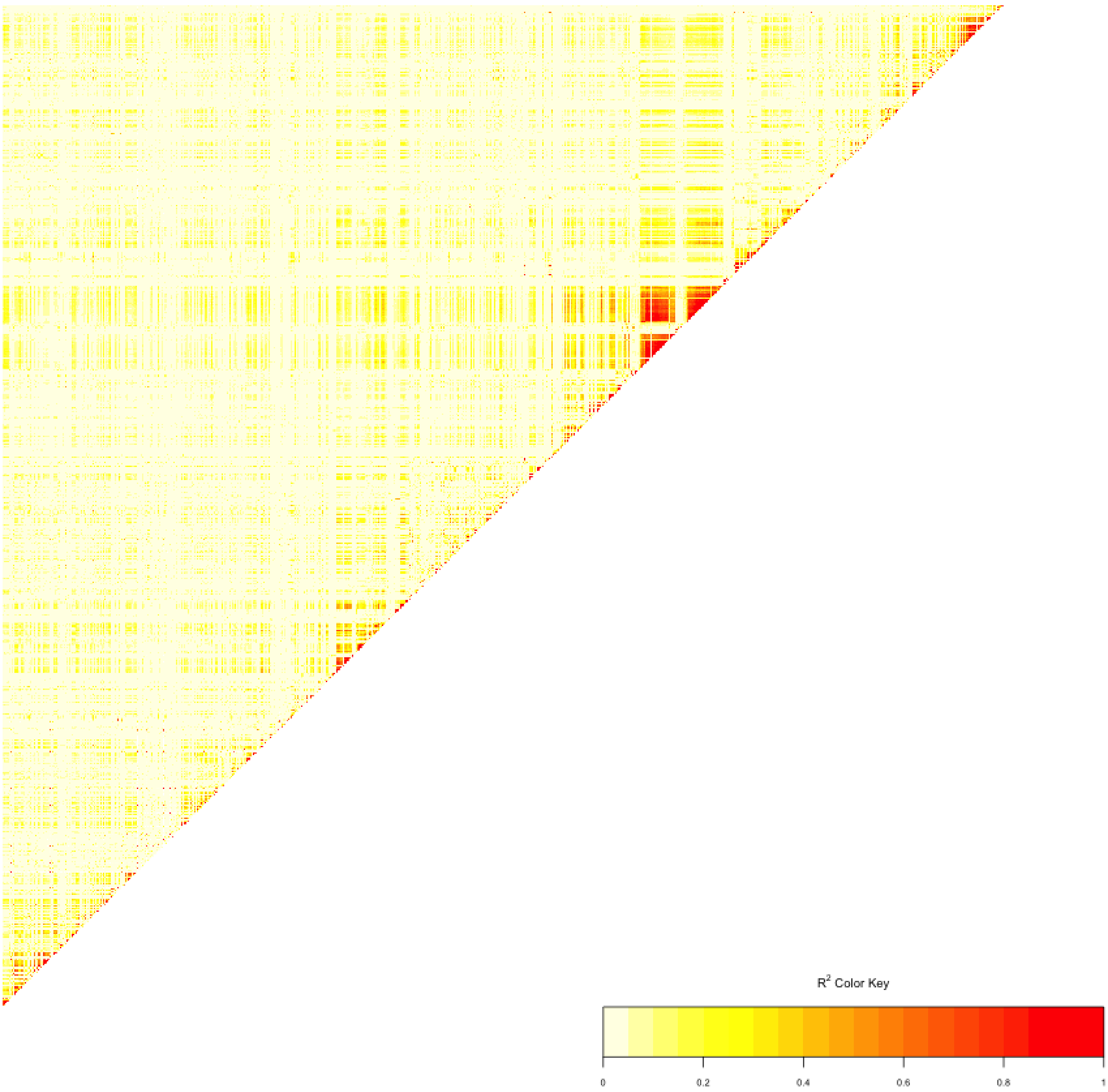

Pairwise LD in r^2 with 936 SNPs in C02 cds range 15_to_6858 out of 6862

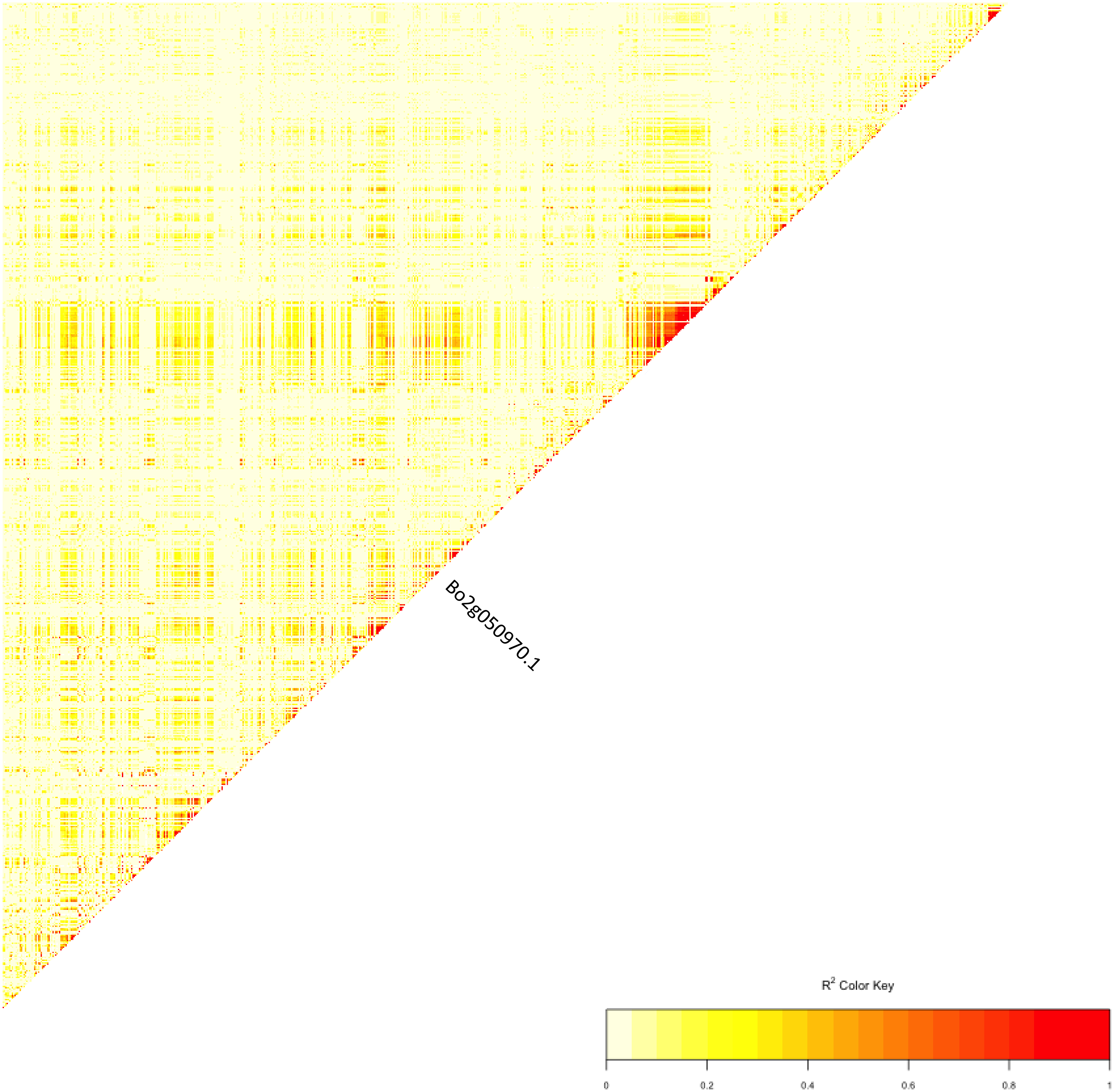

Pairwise LD in r^2 with 1831 SNPs in C03 cds range 20_to_9719 out of 9721

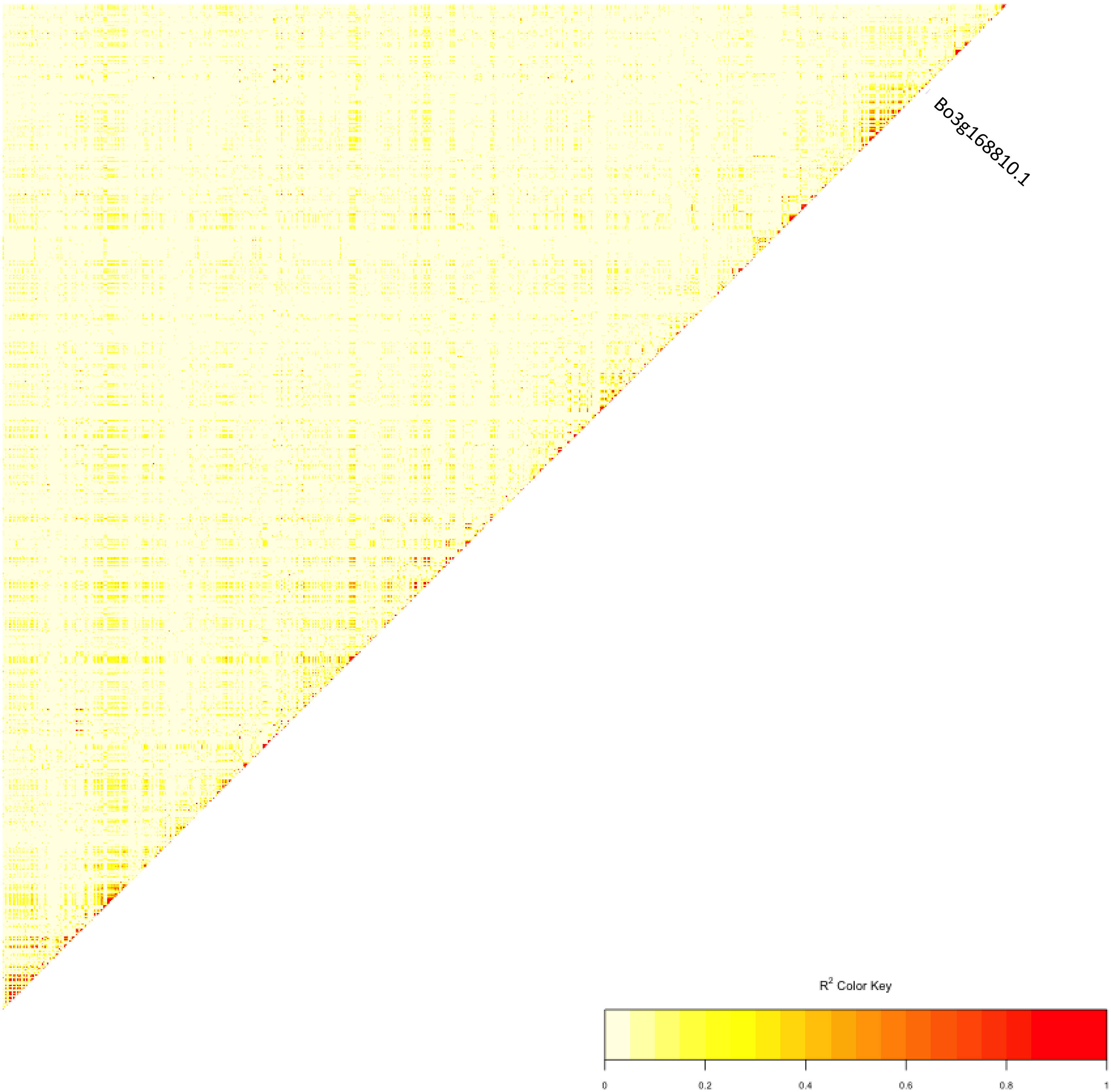

Pairwise LD in r^2 with 1218 SNPs in C04 cds range 4_to_7479 out of 7484

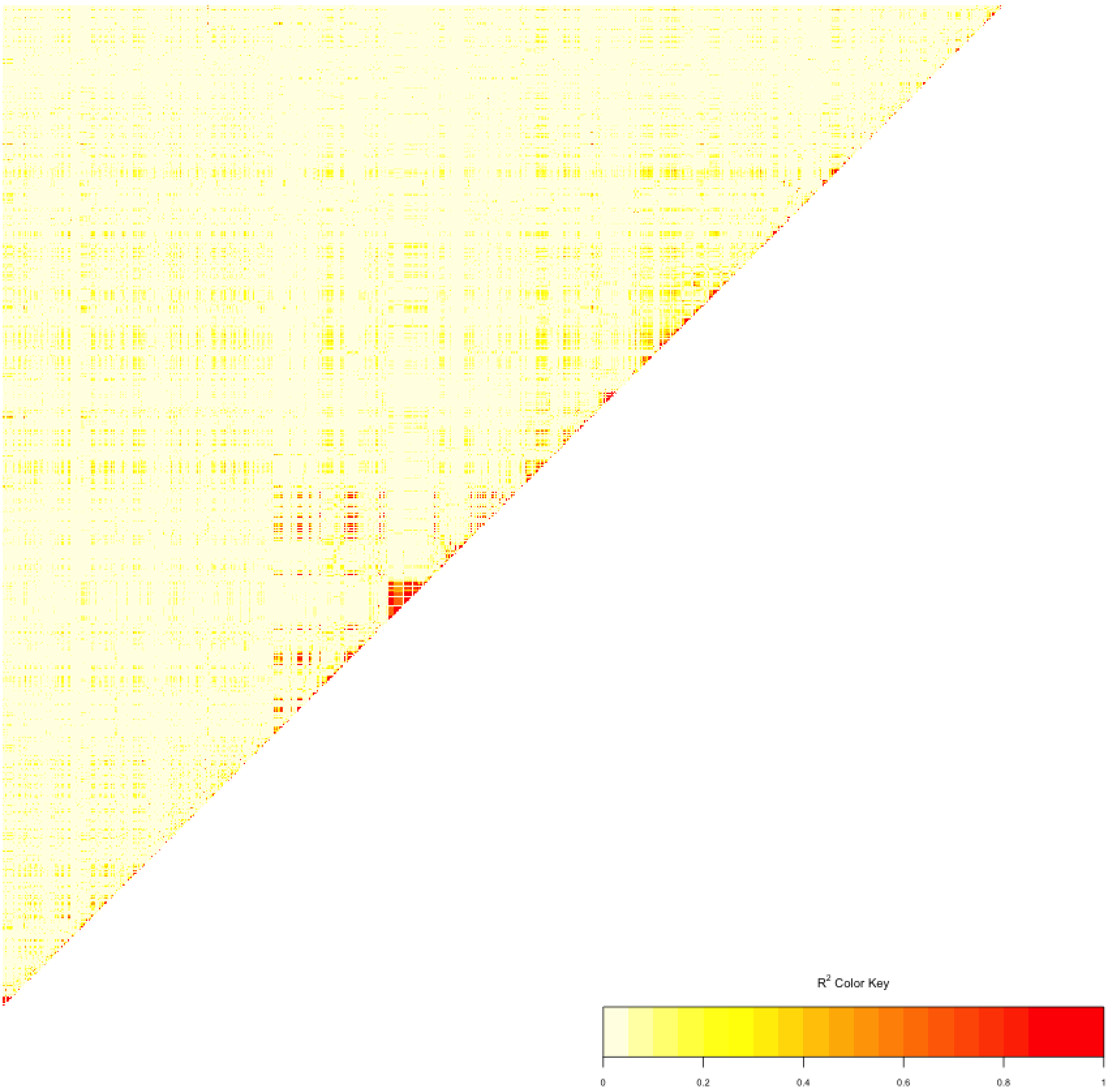

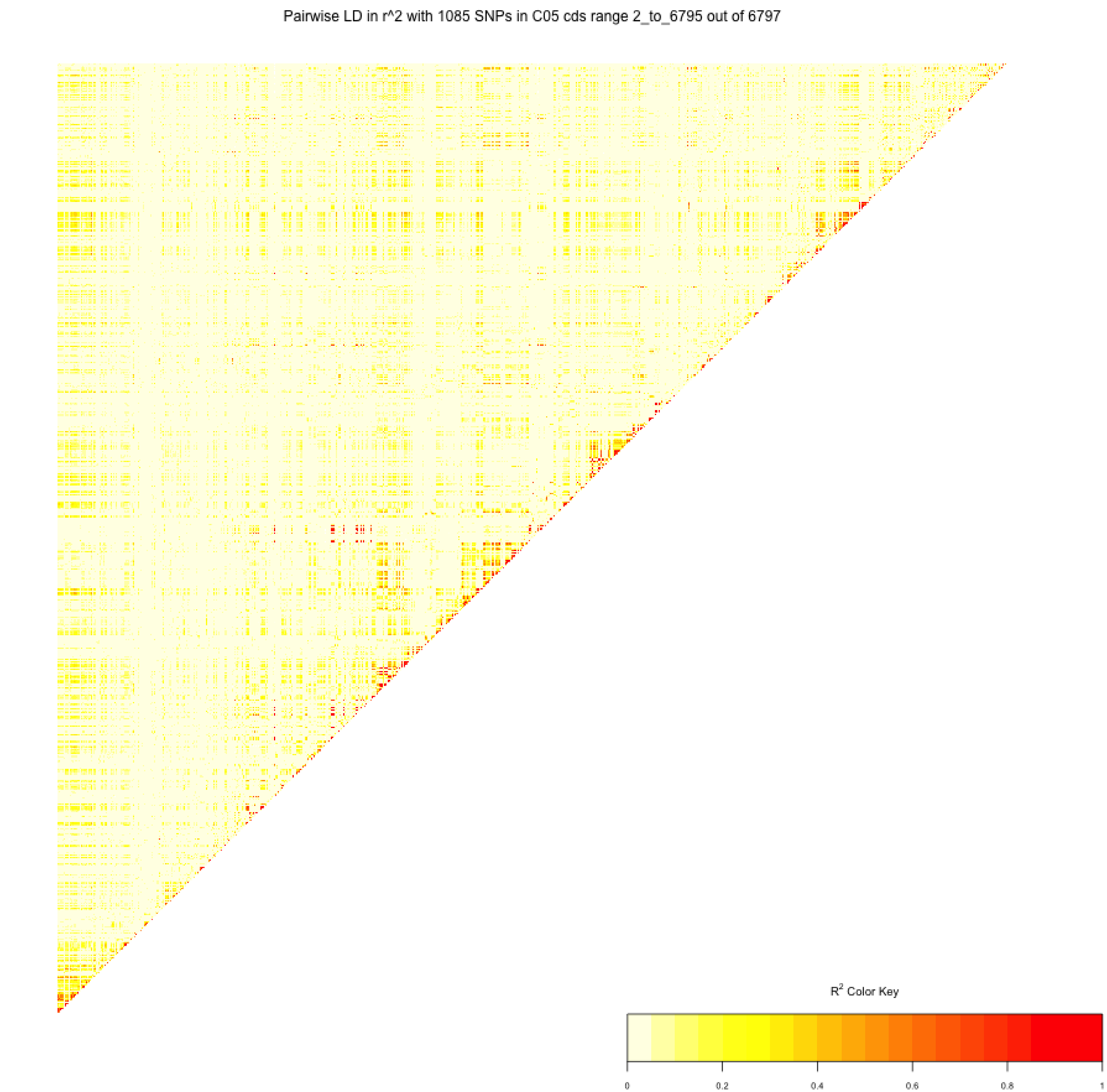
Pairwise LD in r^2 with 1085 SNPs in C05 cds range 2_to_6795 out of 6797
R2 Color Key
0
0.2
0.4
0.6
0.8
1

Pairwise LD in r^2 with 940 SNPs in C06 cds range 21_to_5552 out of 5557

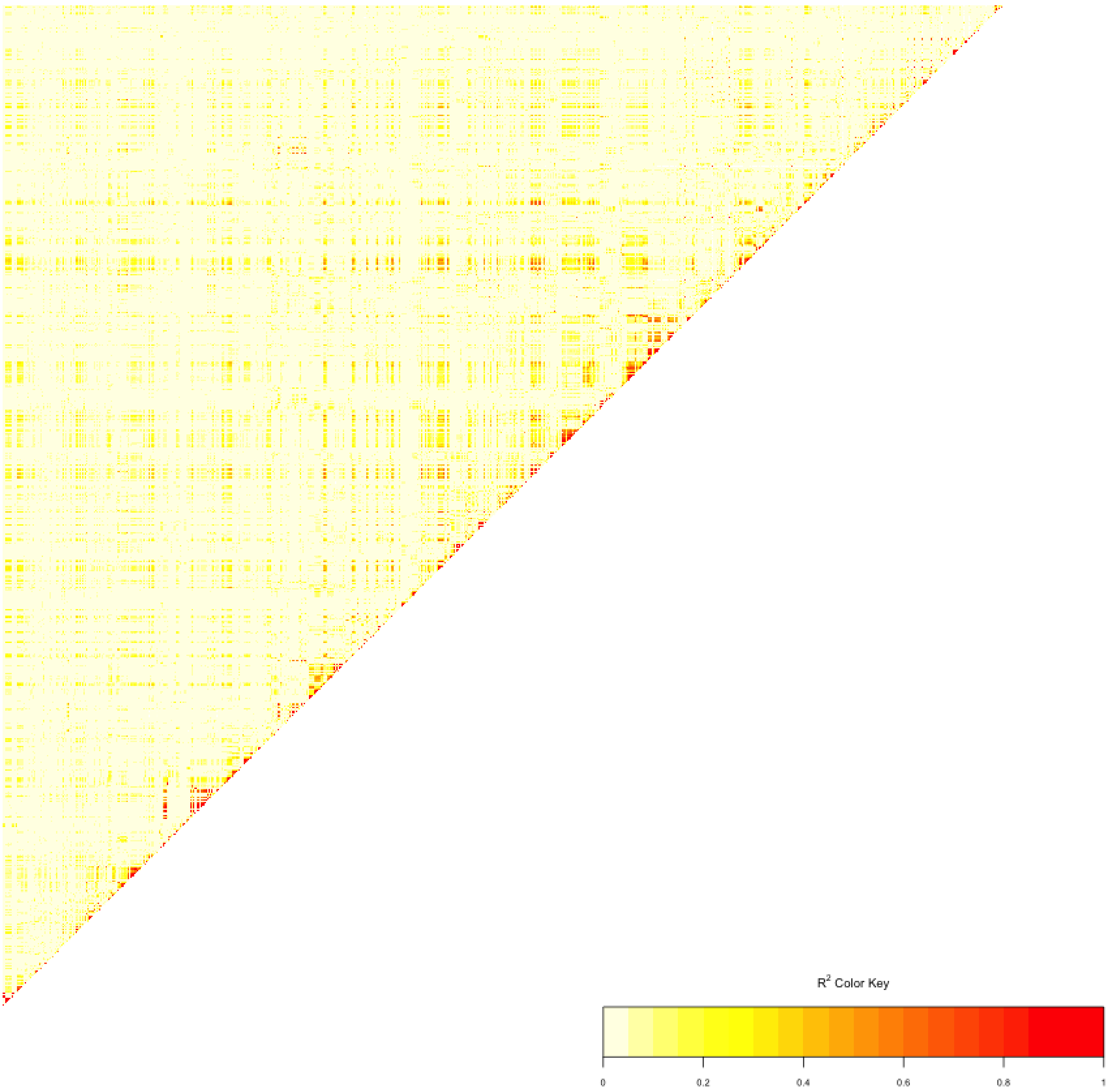

Pairwise LD in r^2 with 1019 SNPs in C07 cds range 52_to_6623 out of 6623

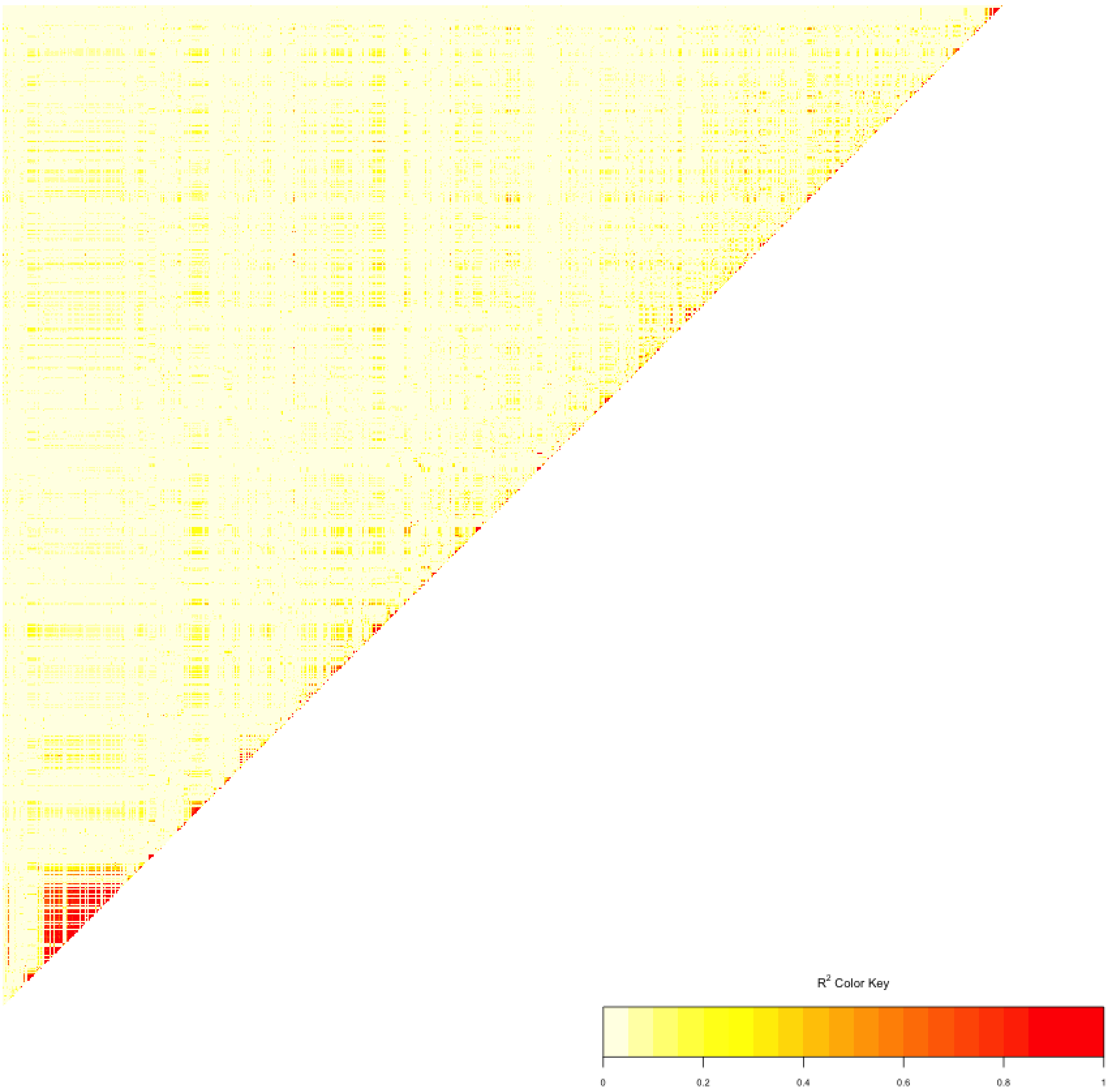

Pairwise LD in r^2 with 1013 SNPs in C08 cds range 29_to_6414 out of 6418

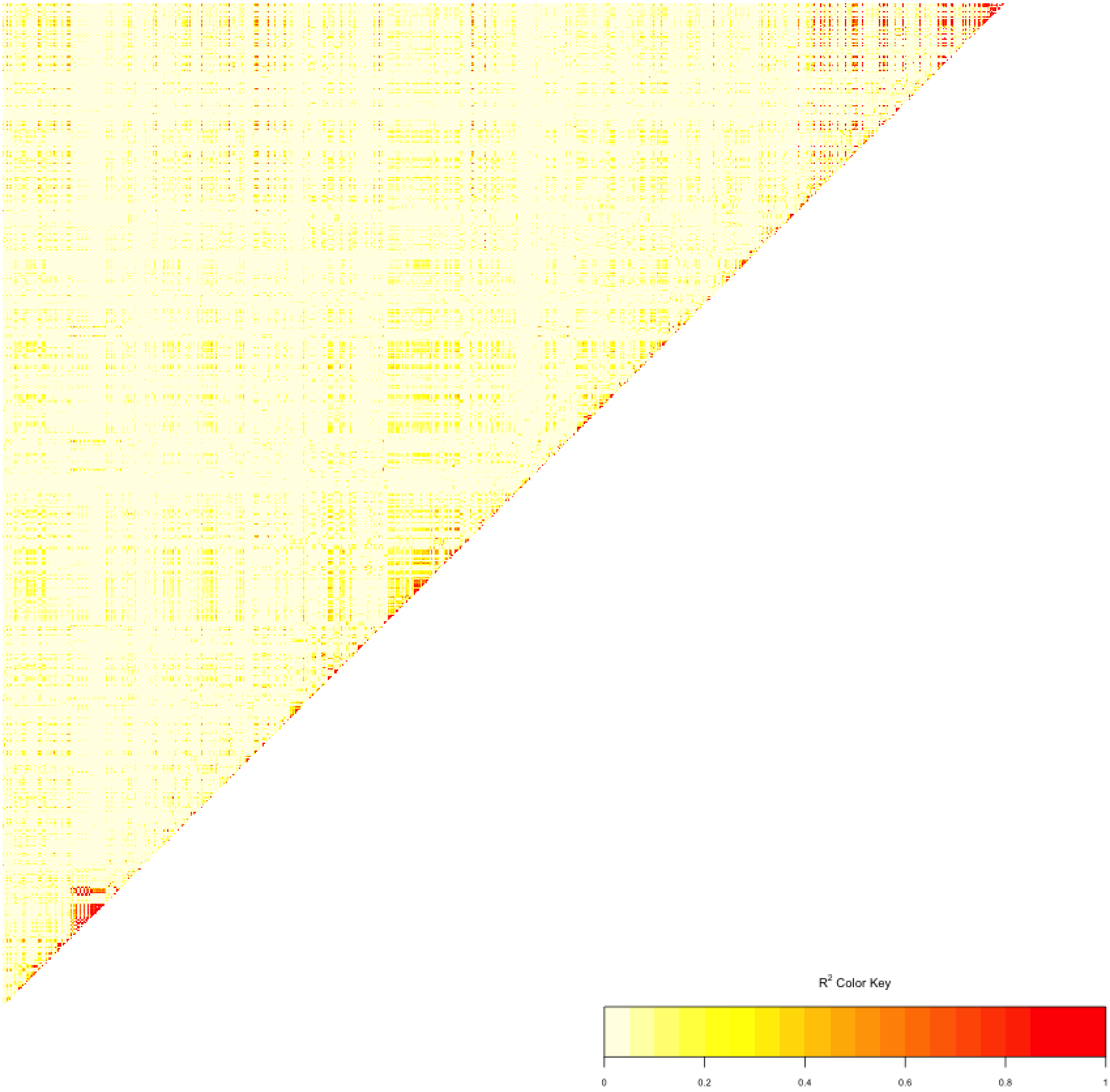

Pairwise LD in r^2 with 1215 SNPs in C09 cds range 12_to_7631 out of 7635

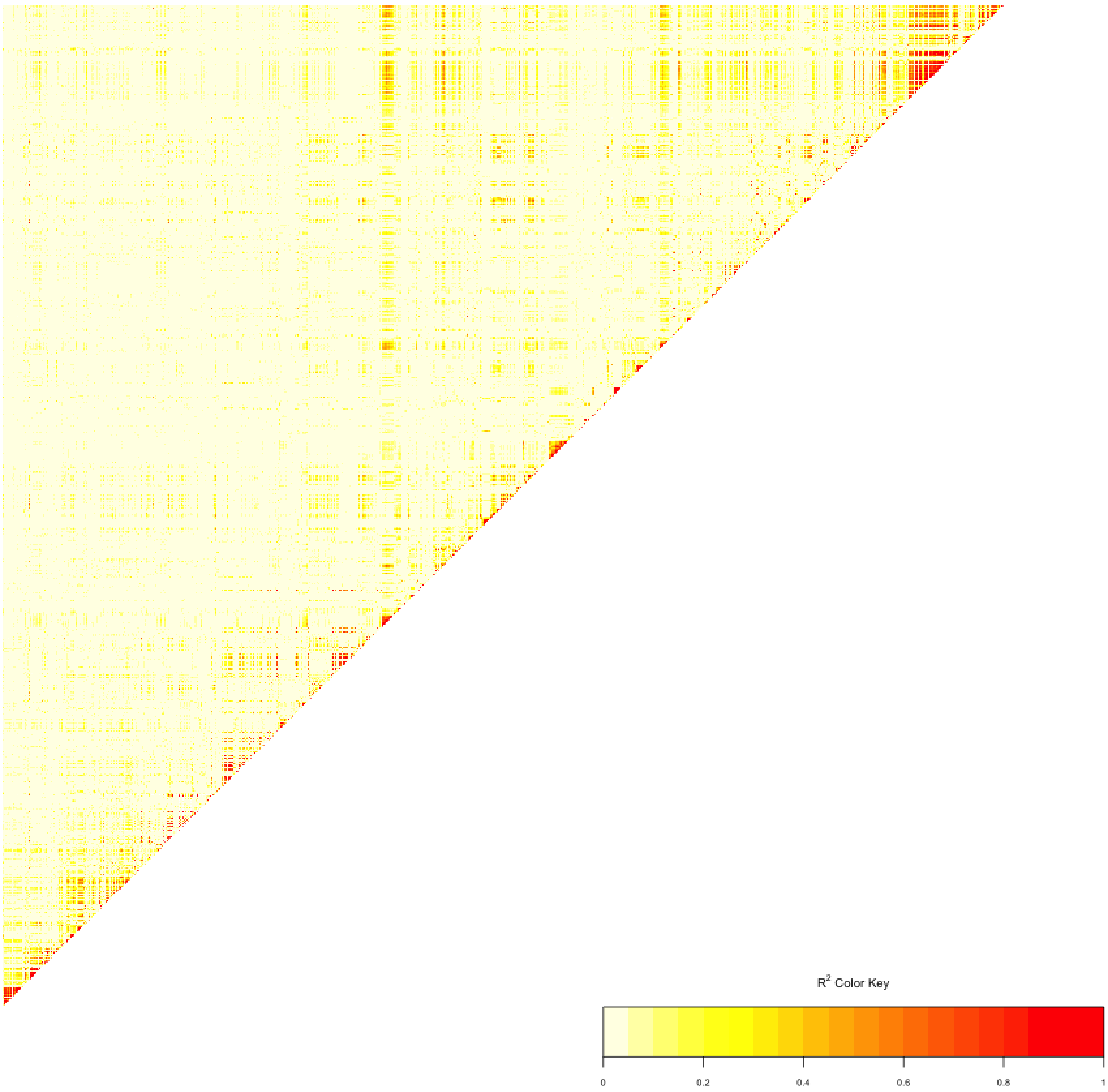

Supplement: Supplementary file 1 — Figure S1. Genome‐wide linkage disequilibrium analysis for the RIPR diversity panel. [file TPJ-93-181-s001.pdf]
