# Supplementary figures and images for: Validation of an updated Associative Transcriptomics platform for the polyploid crop species Brassica napus by dissection of the genetic architecture of erucic acid and tocopherol isoform variation in seeds
Source: Plant J. 2017 Dec 2;93(1):181–92. doi: 10.1111/tpj.13767 (PMC5767744; doi:10.1111/tpj.13767)

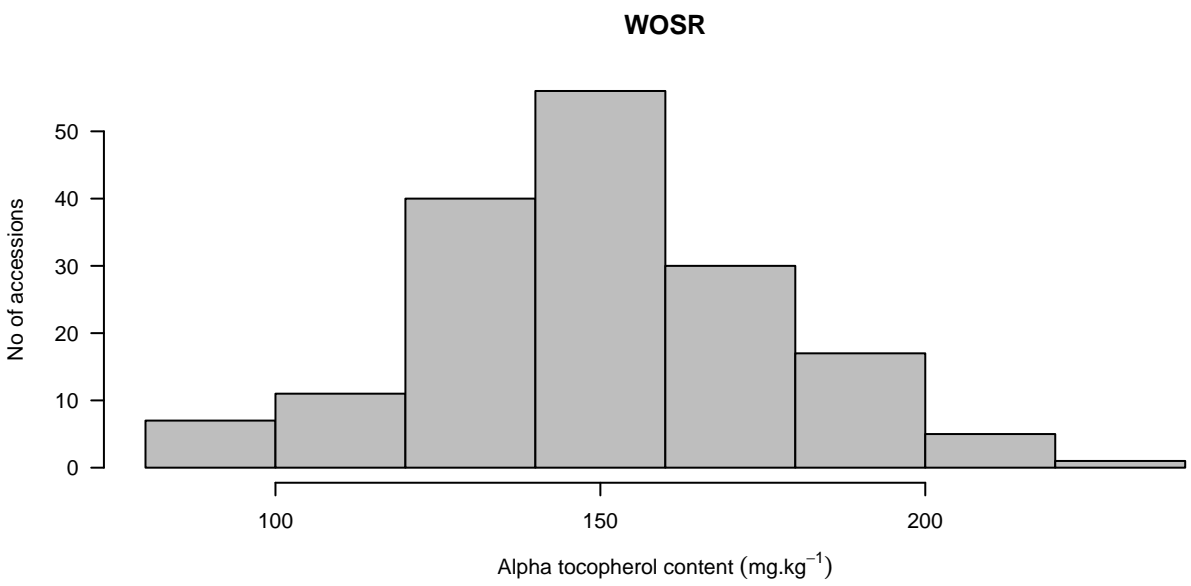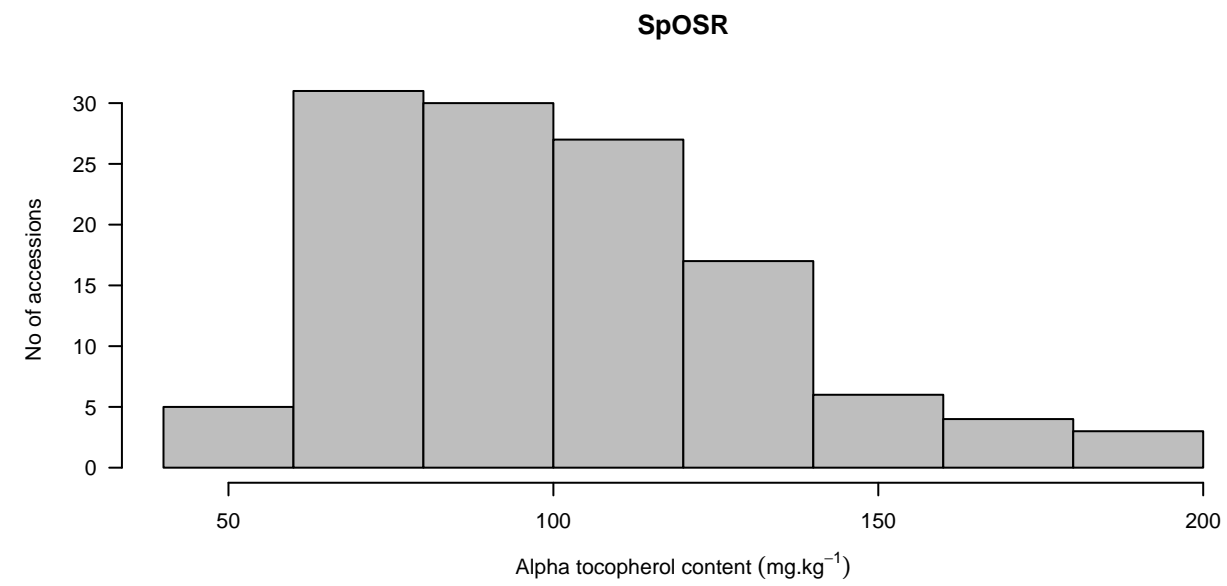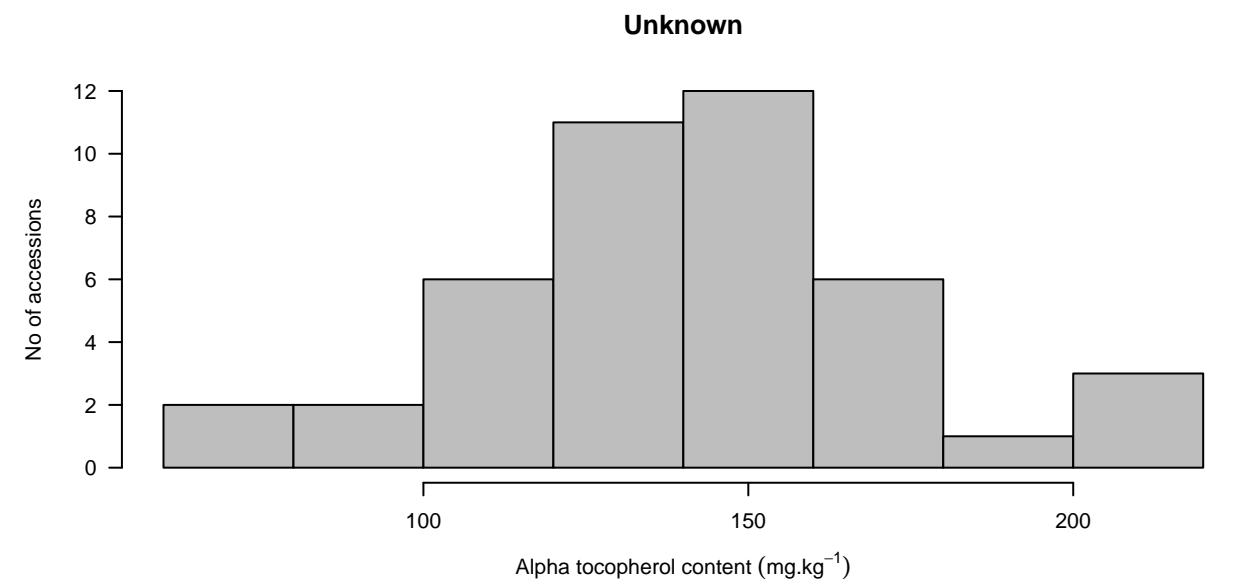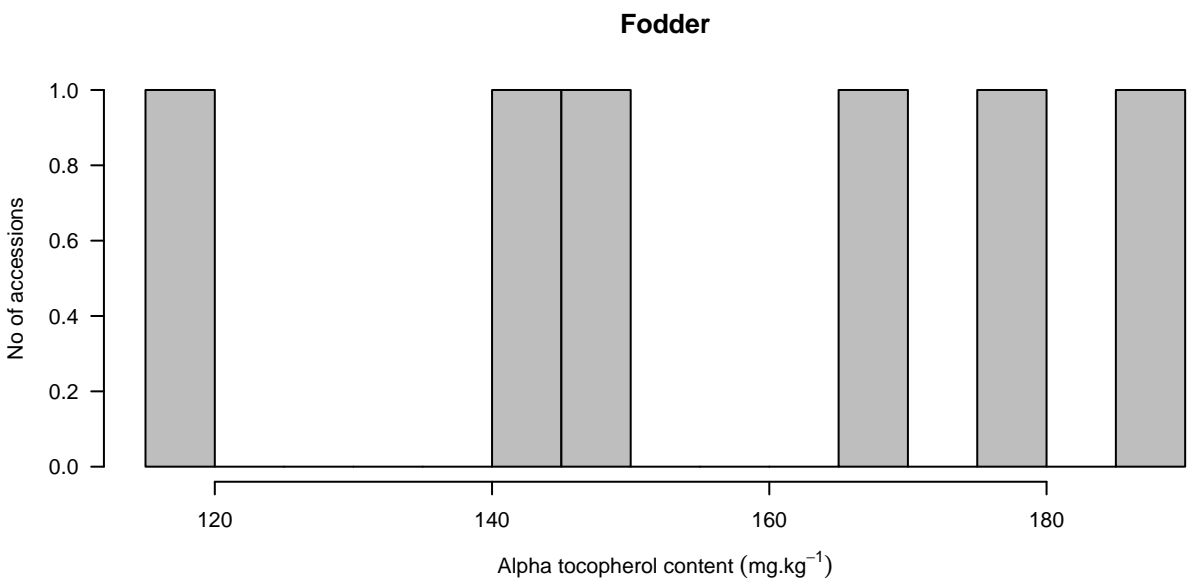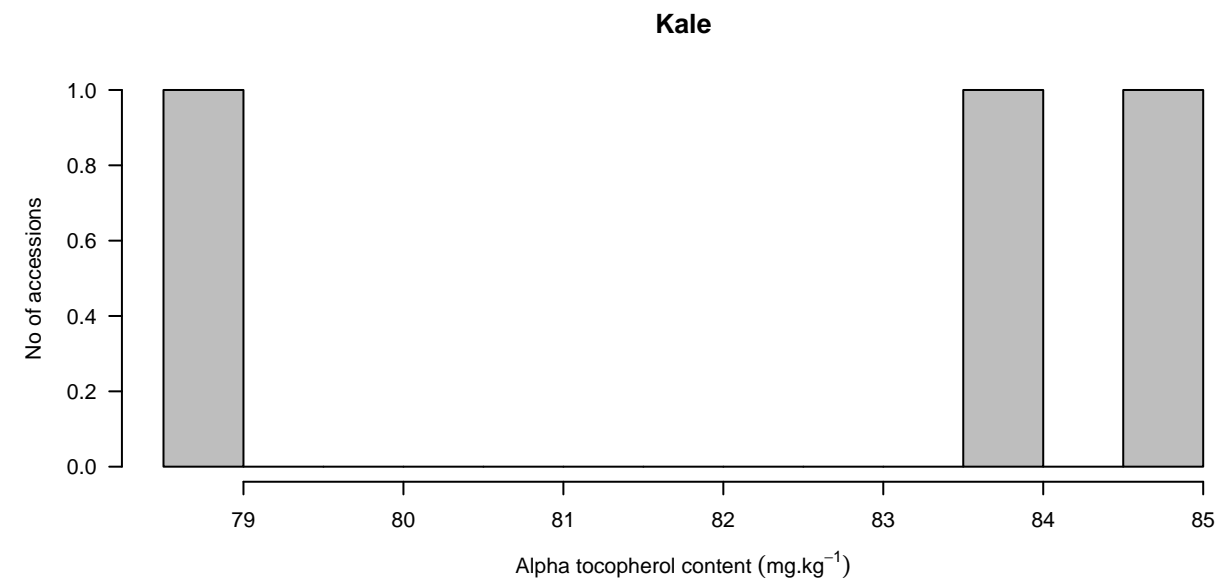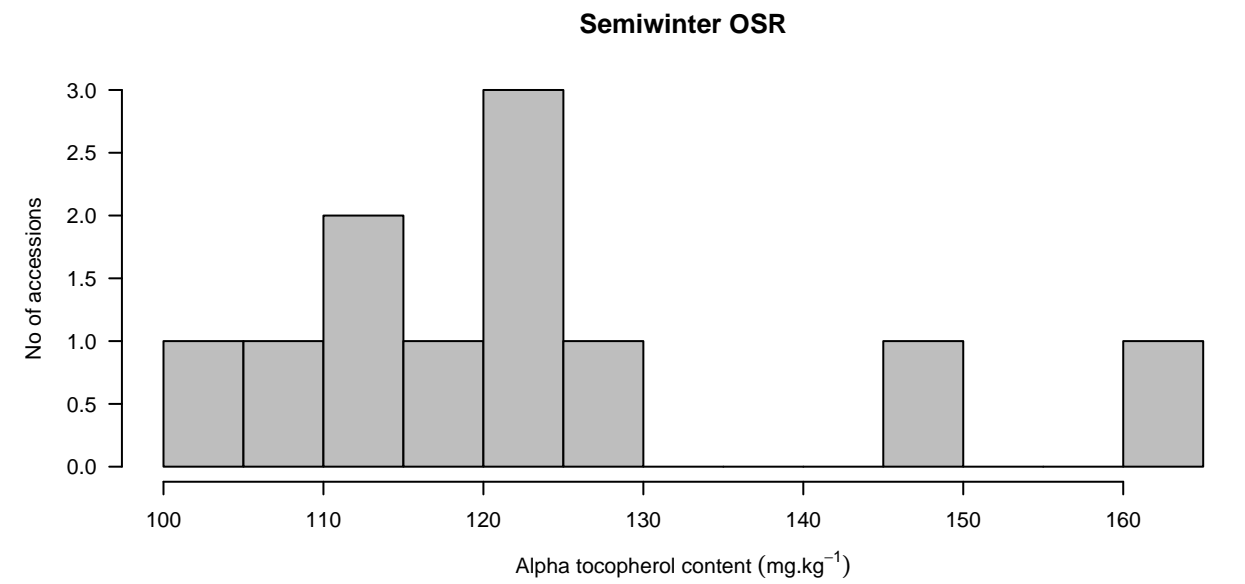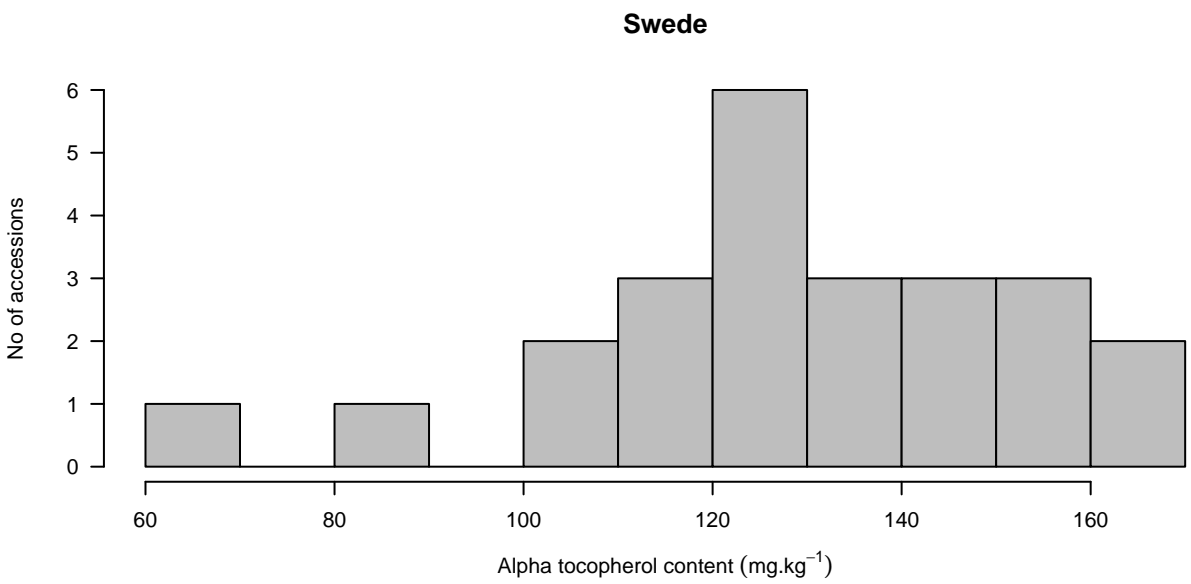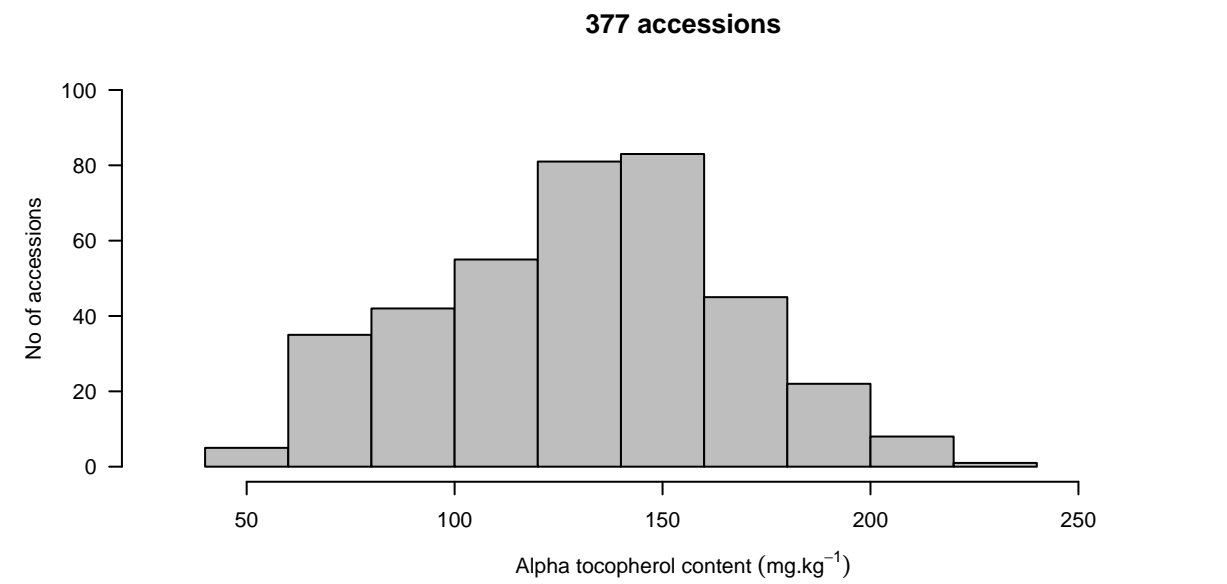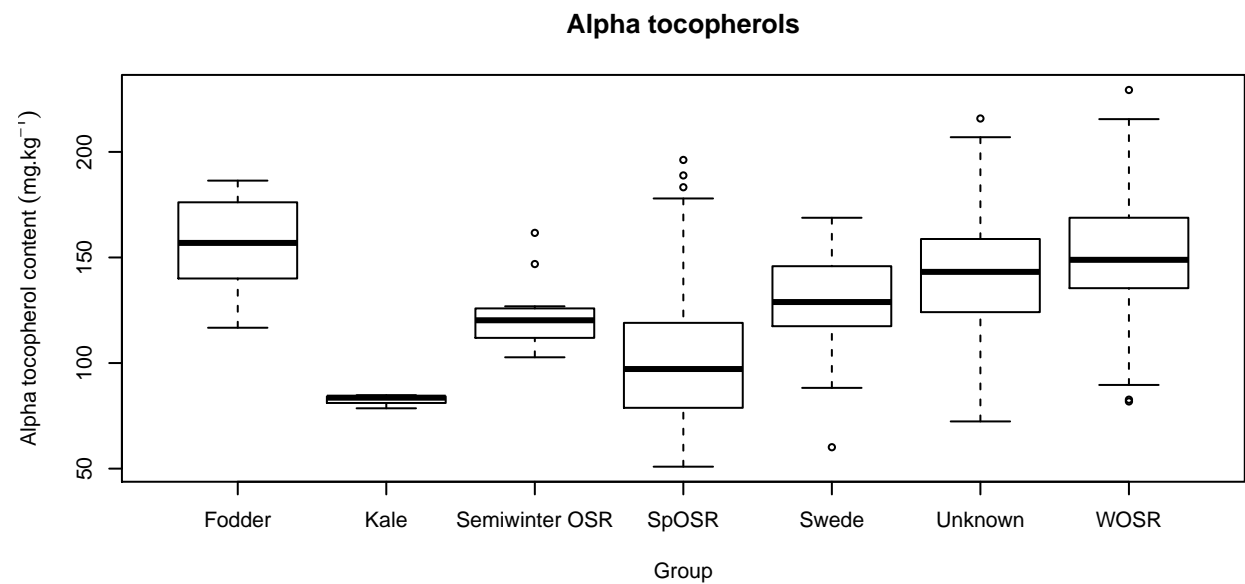

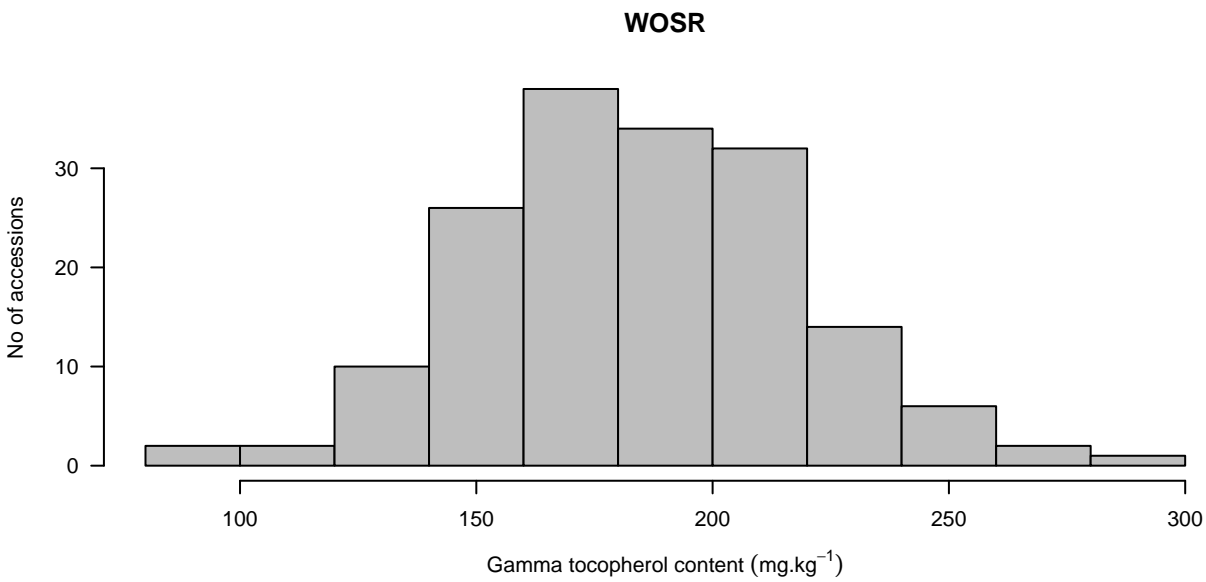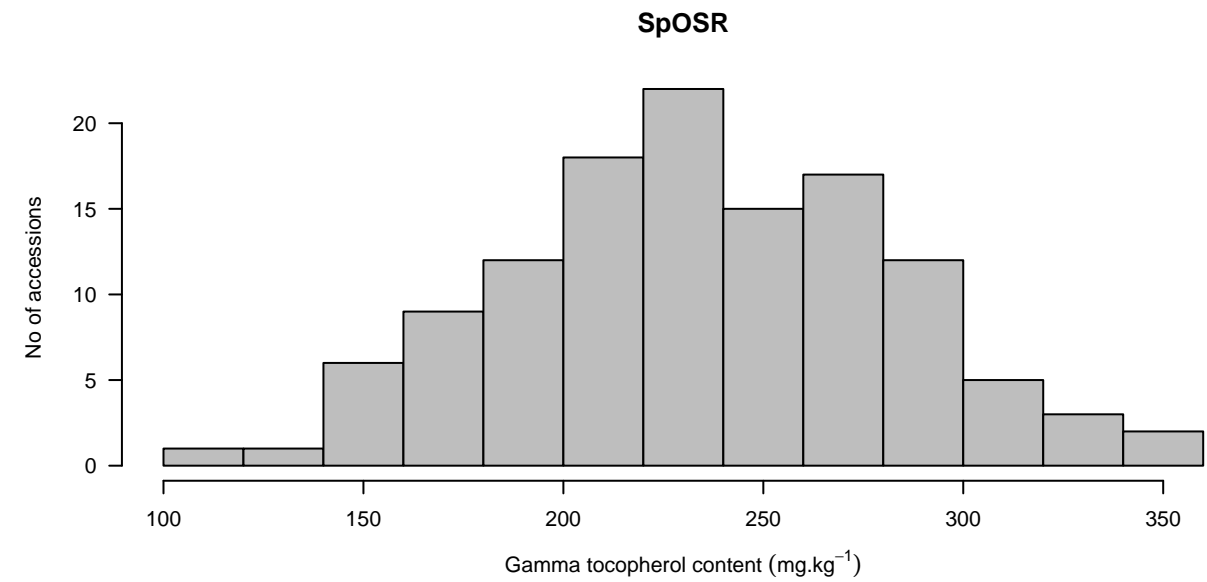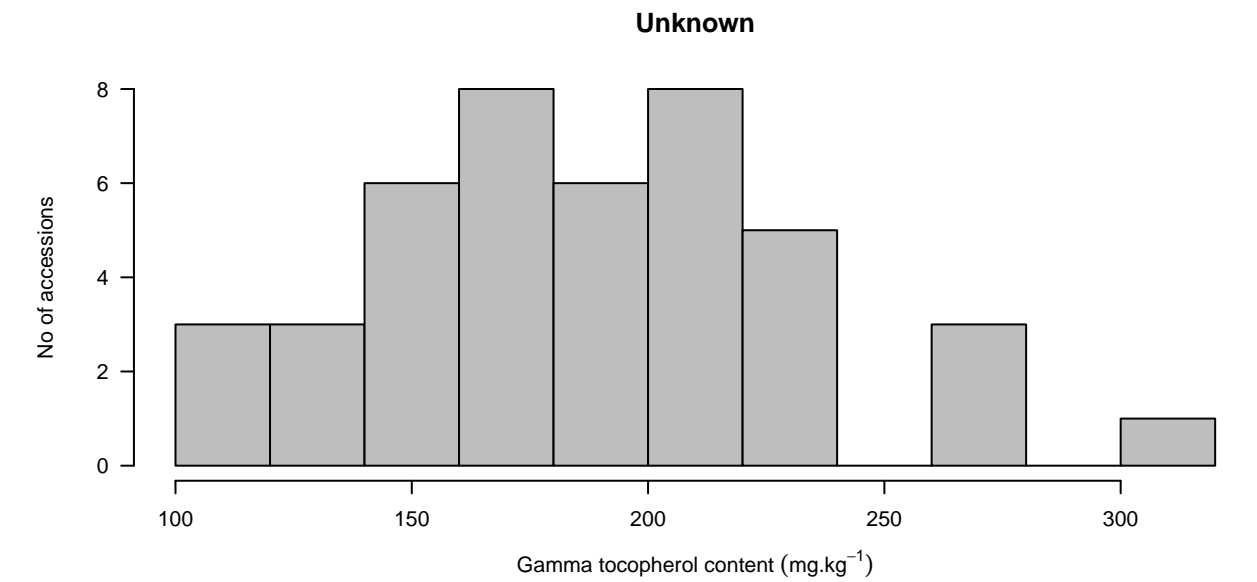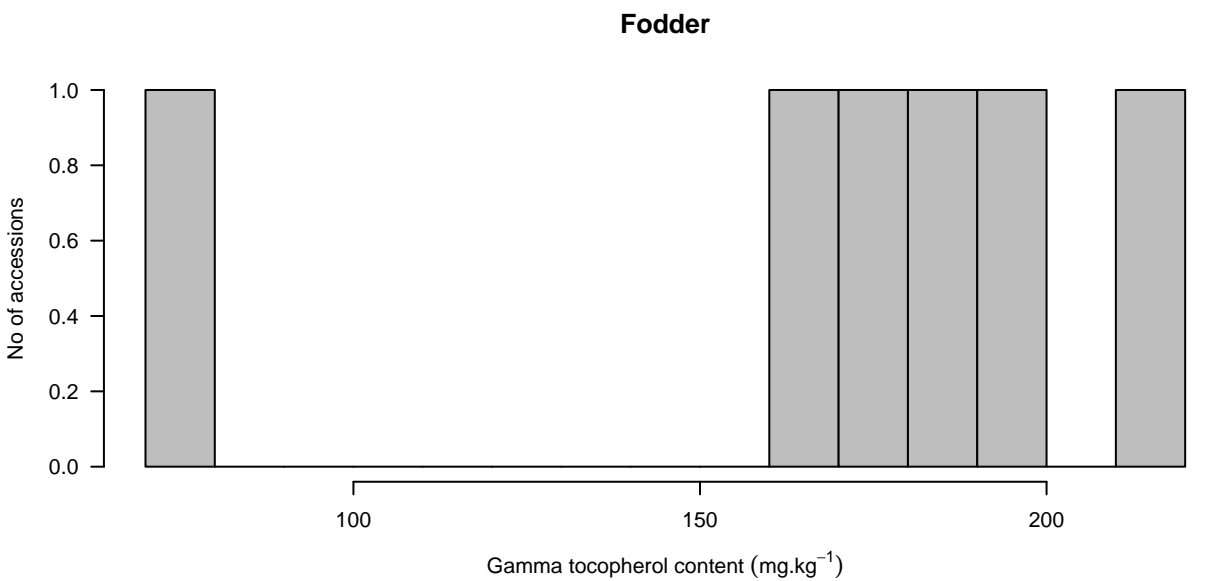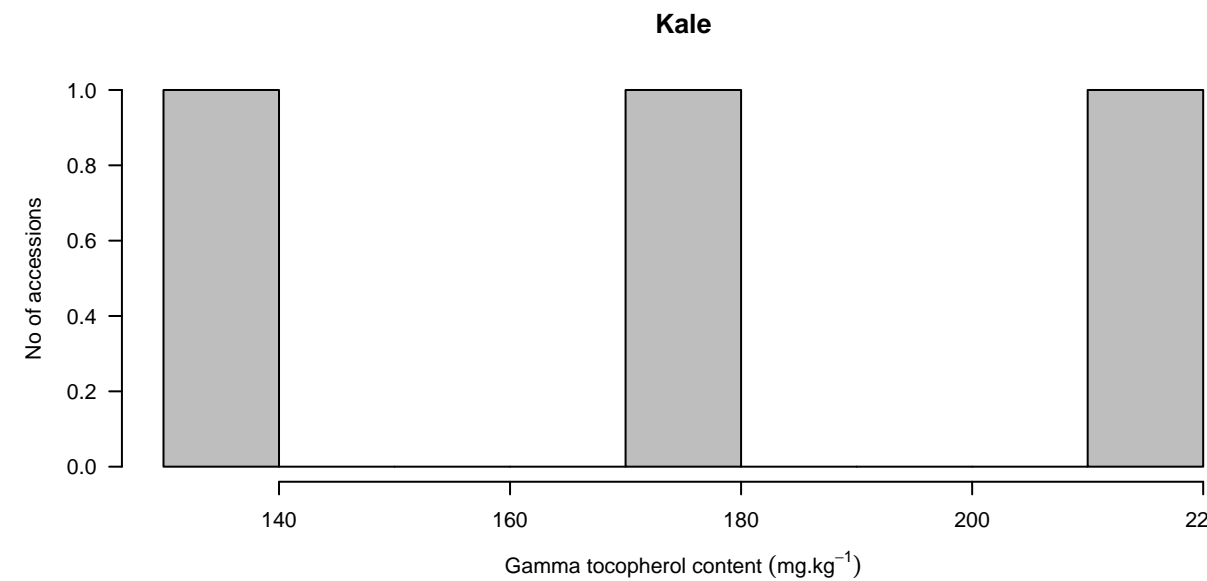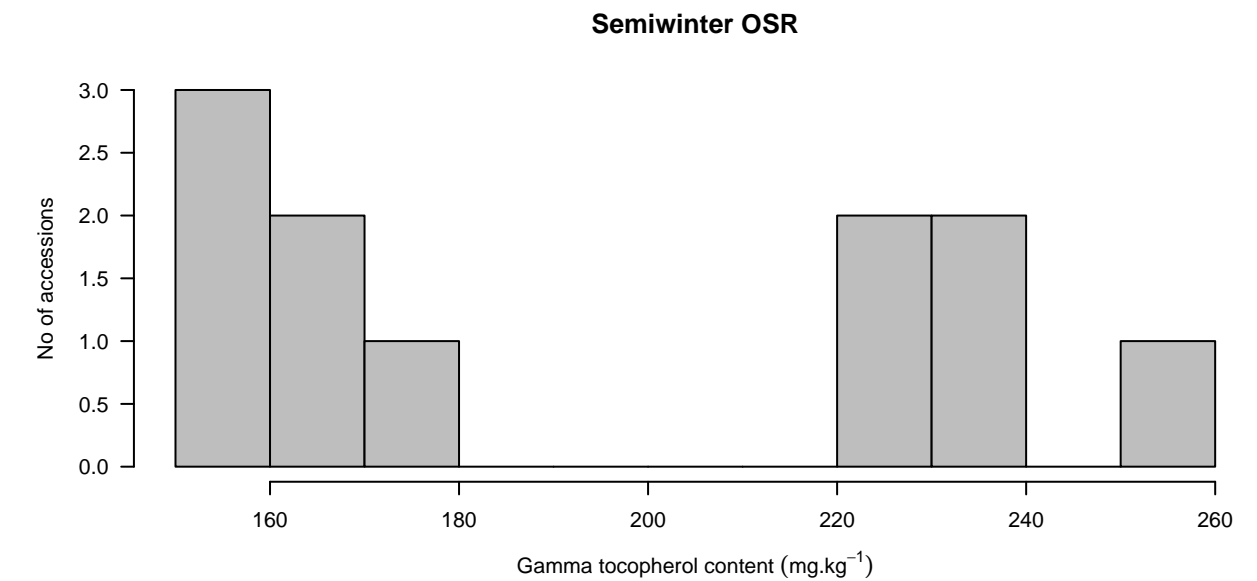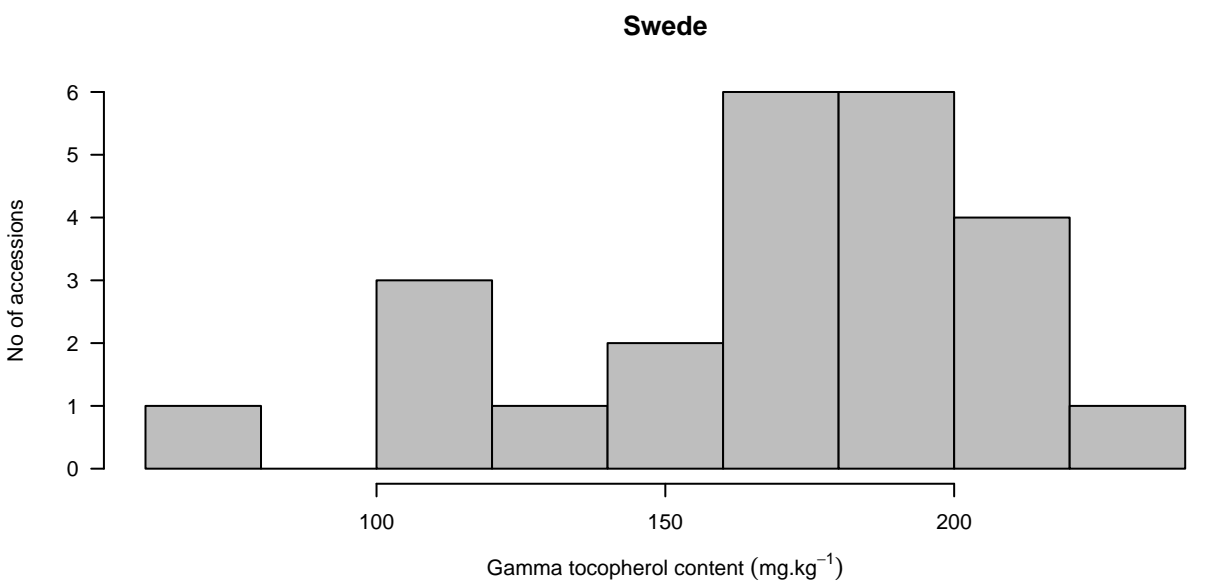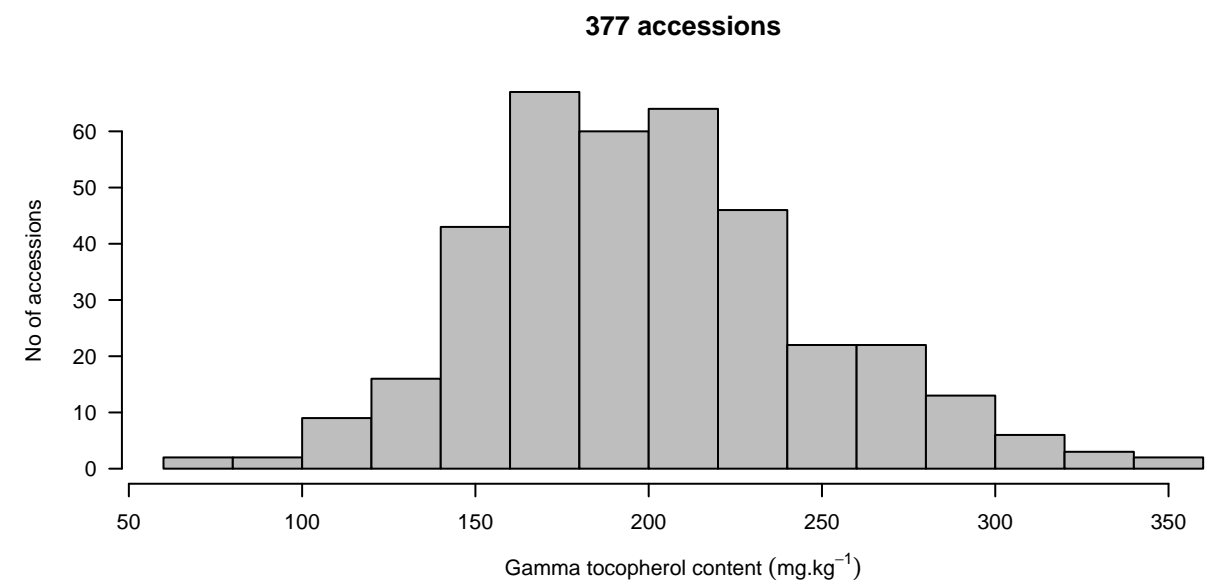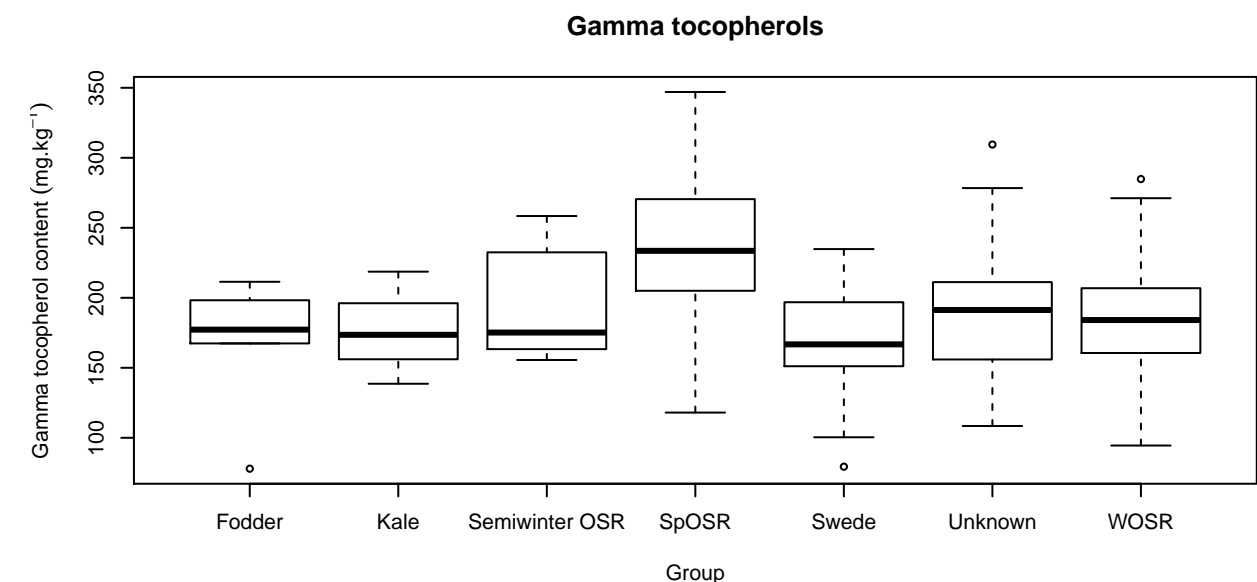

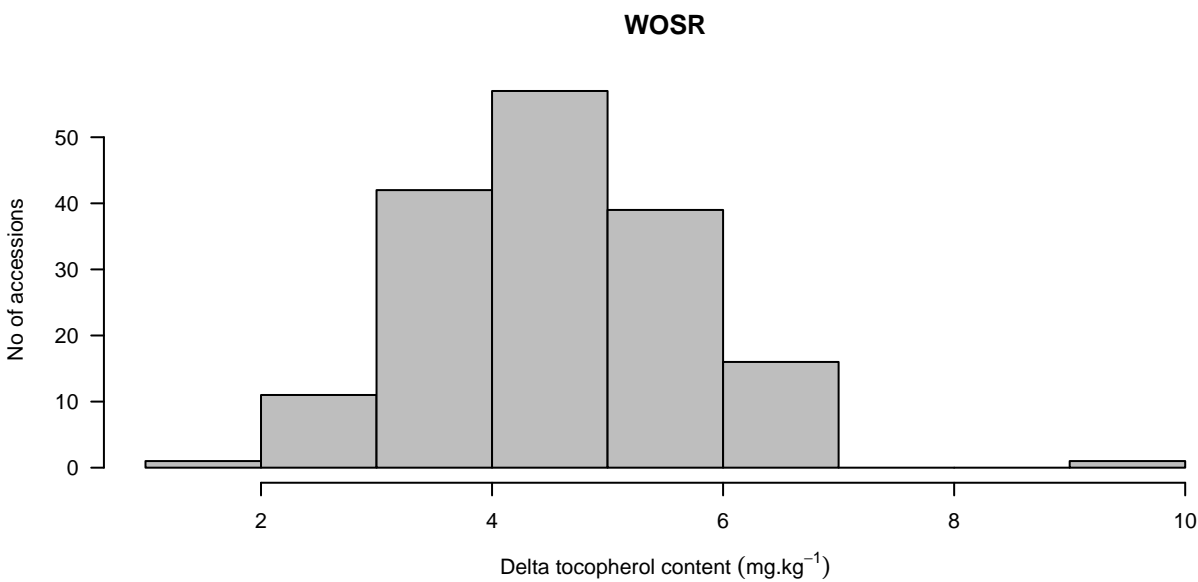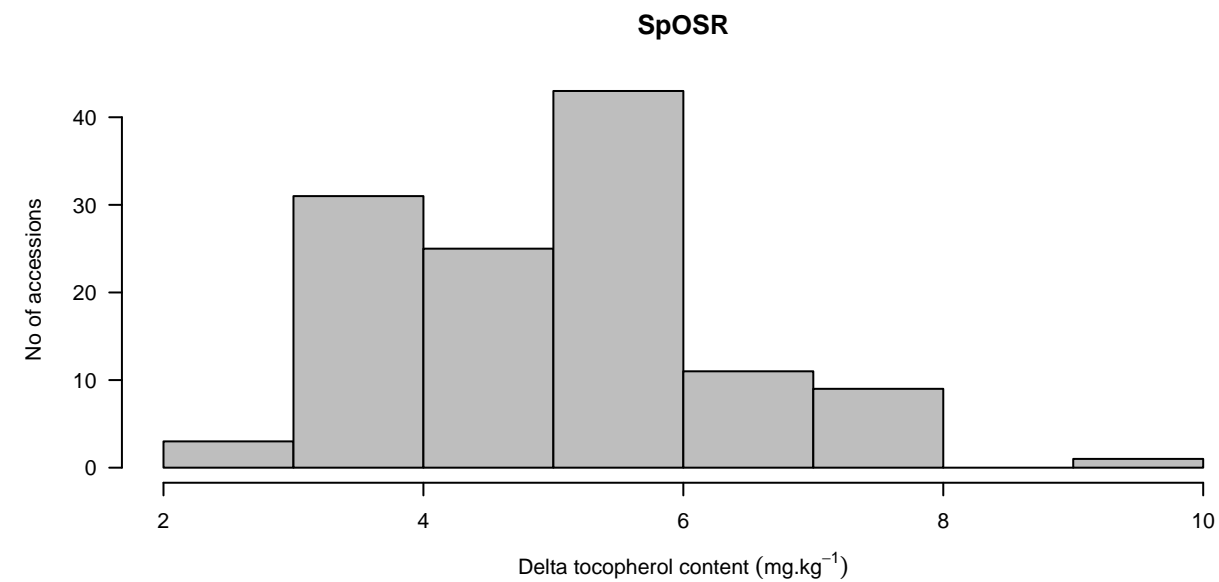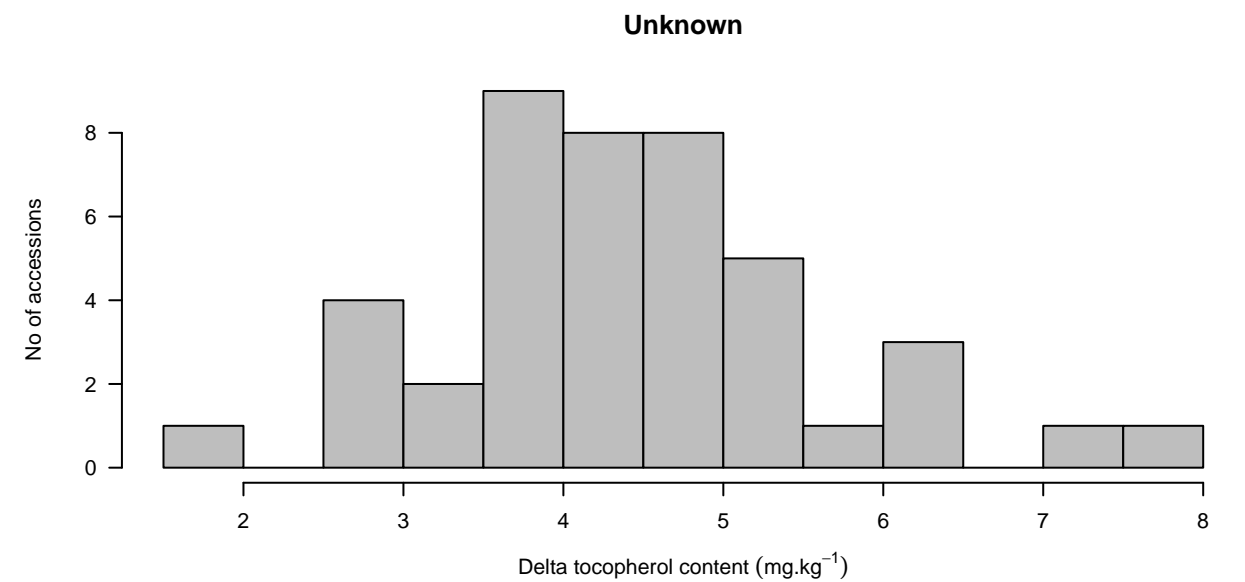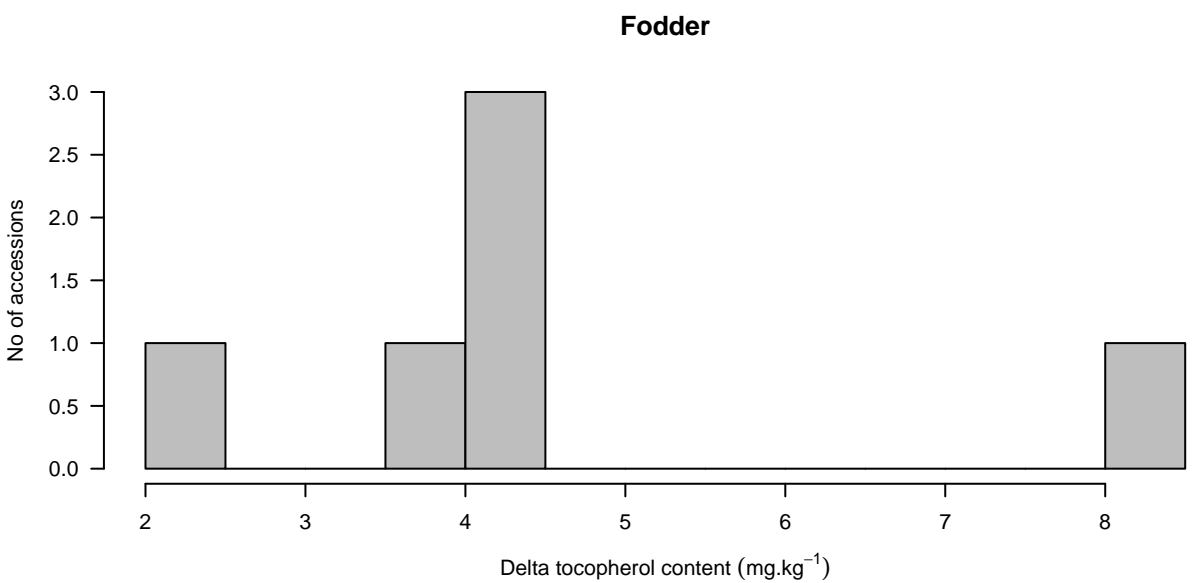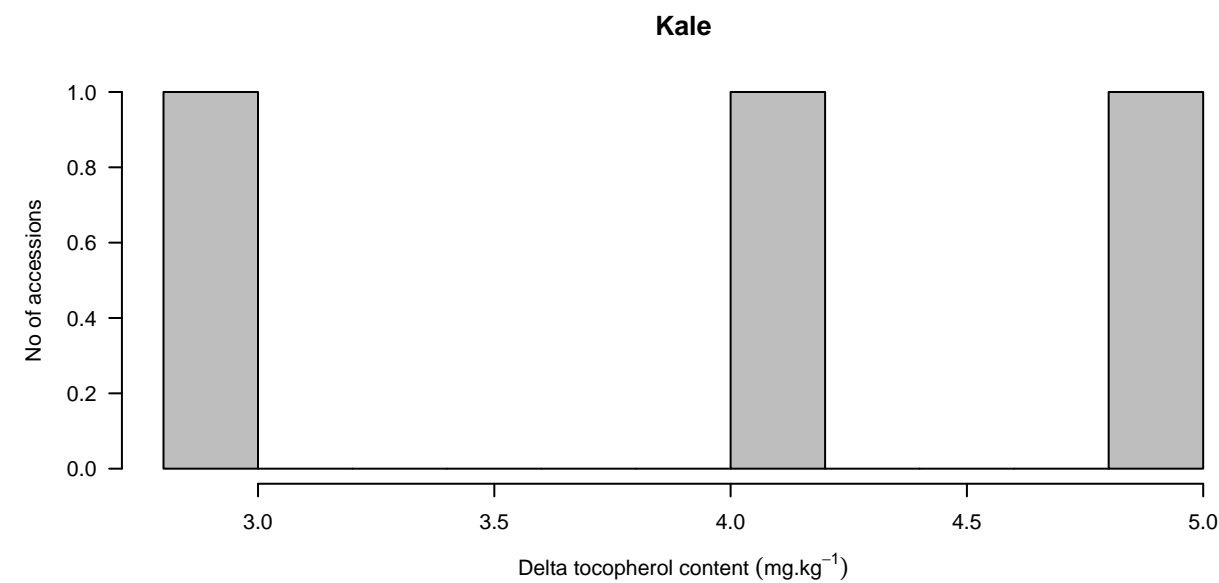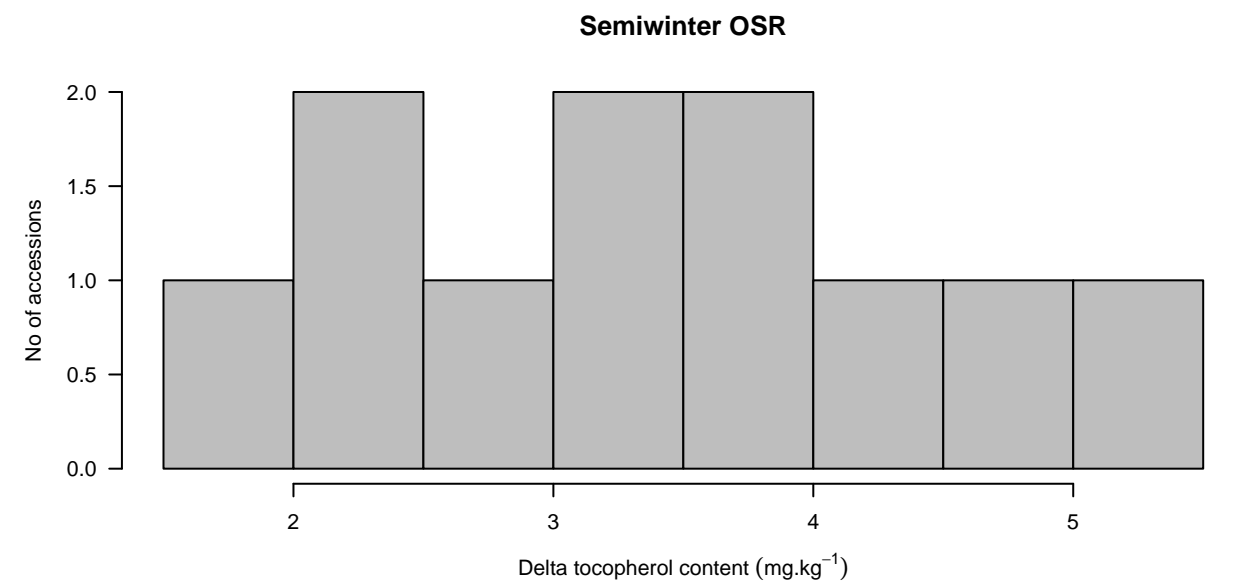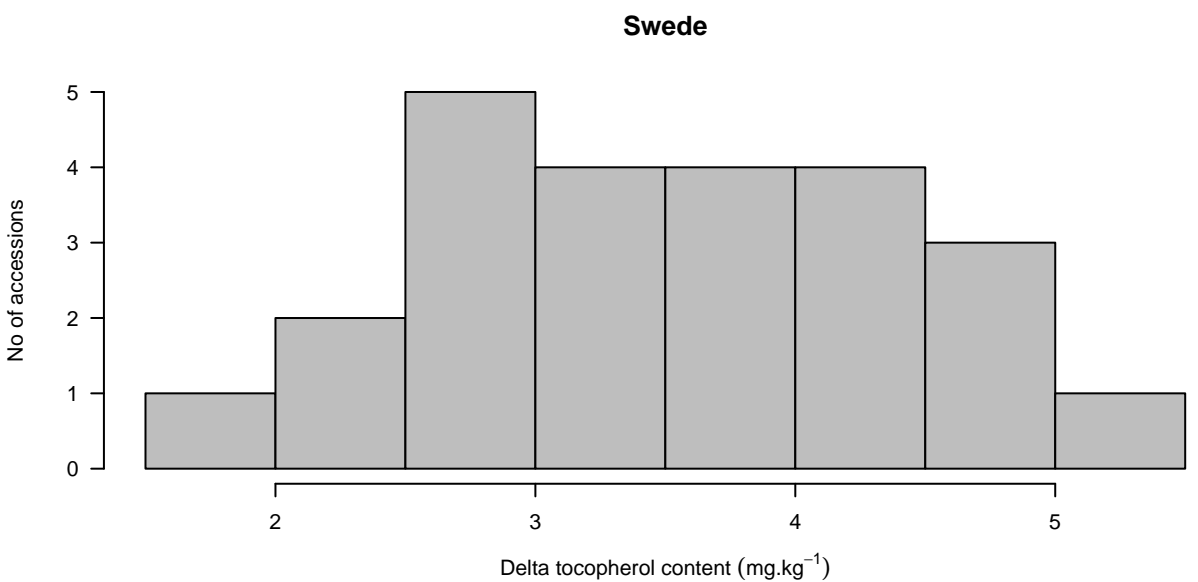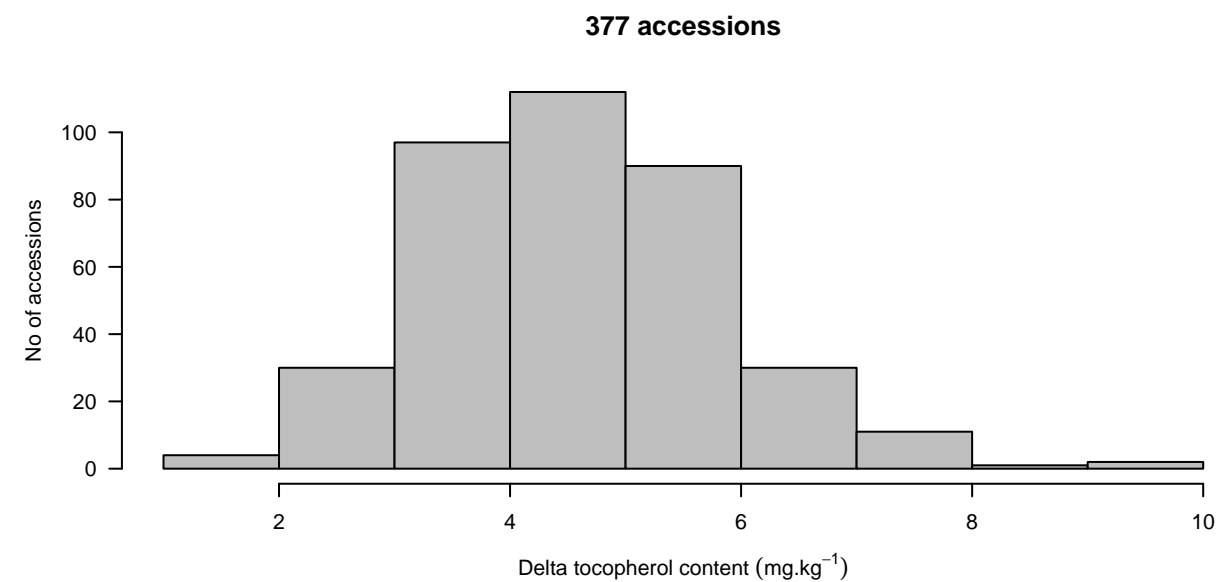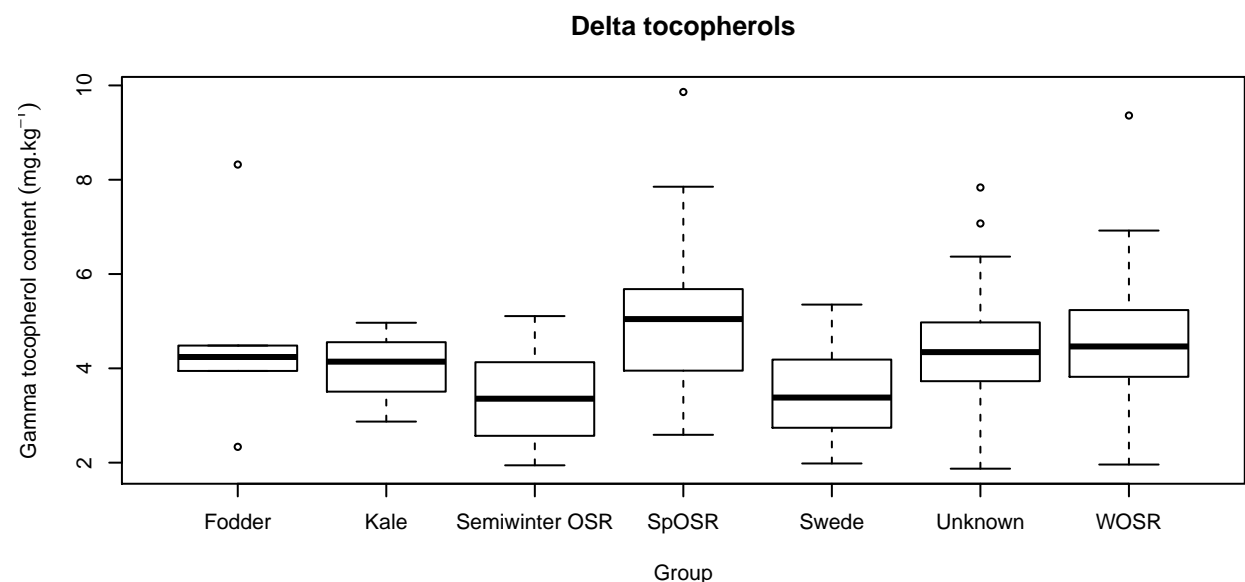

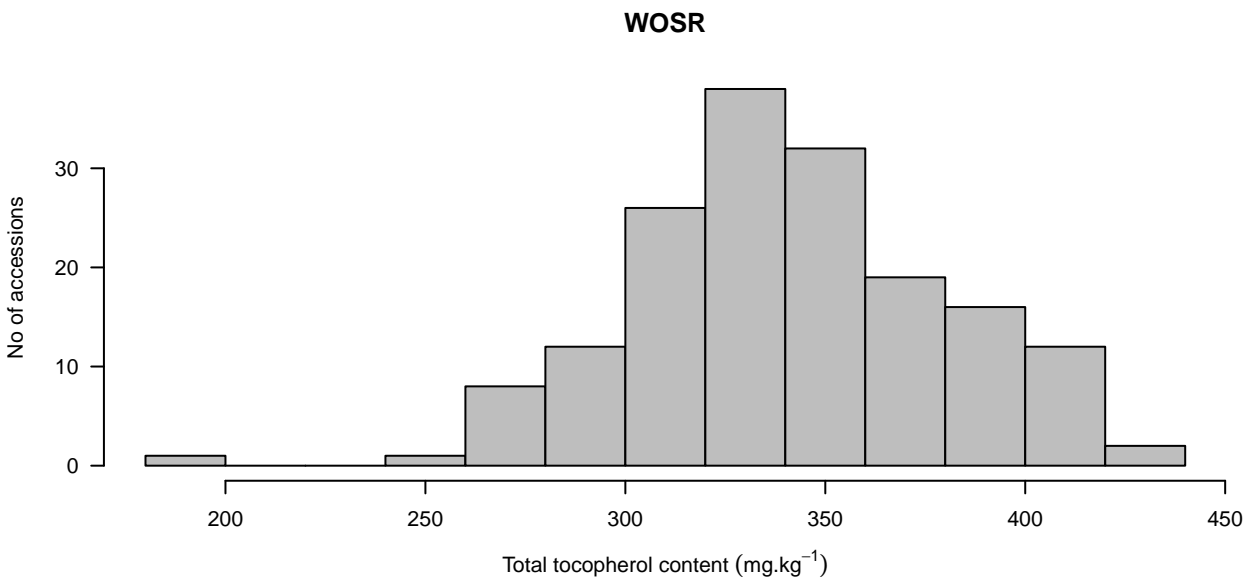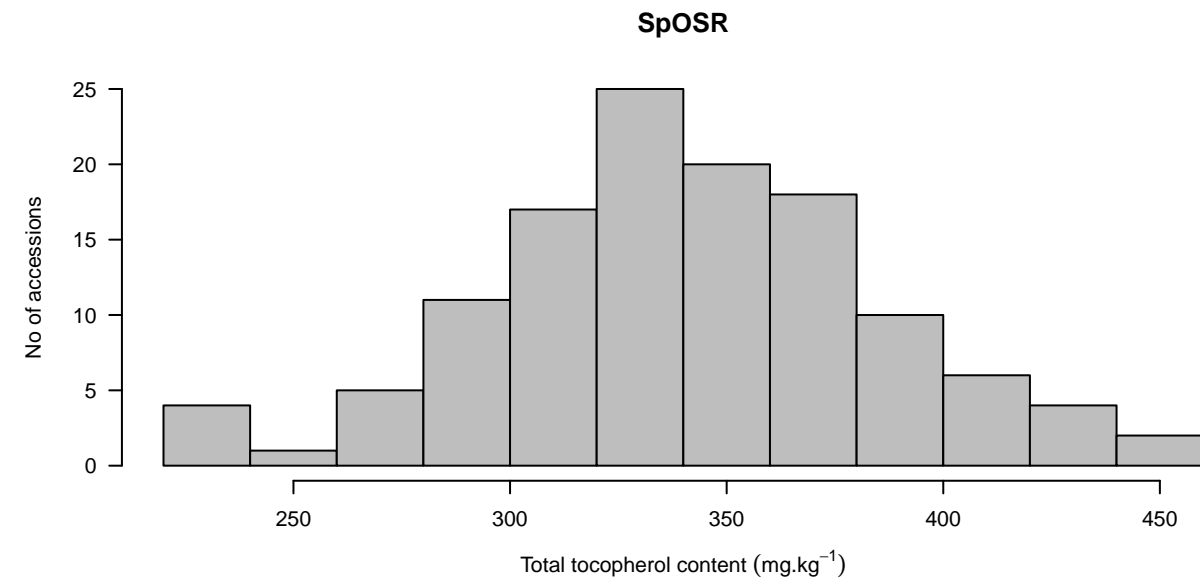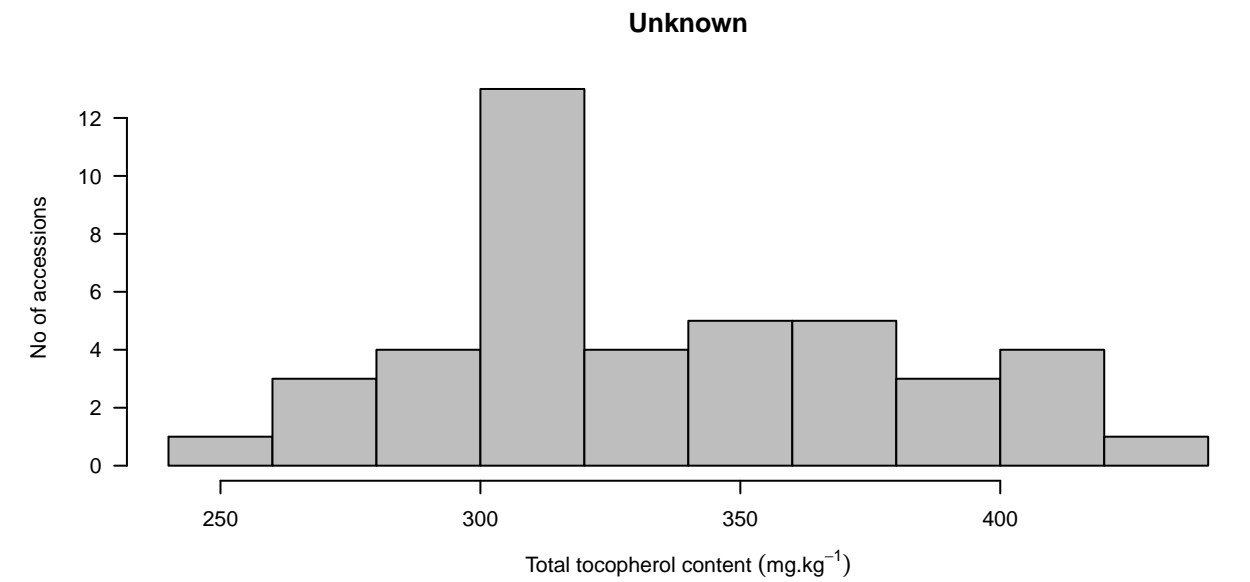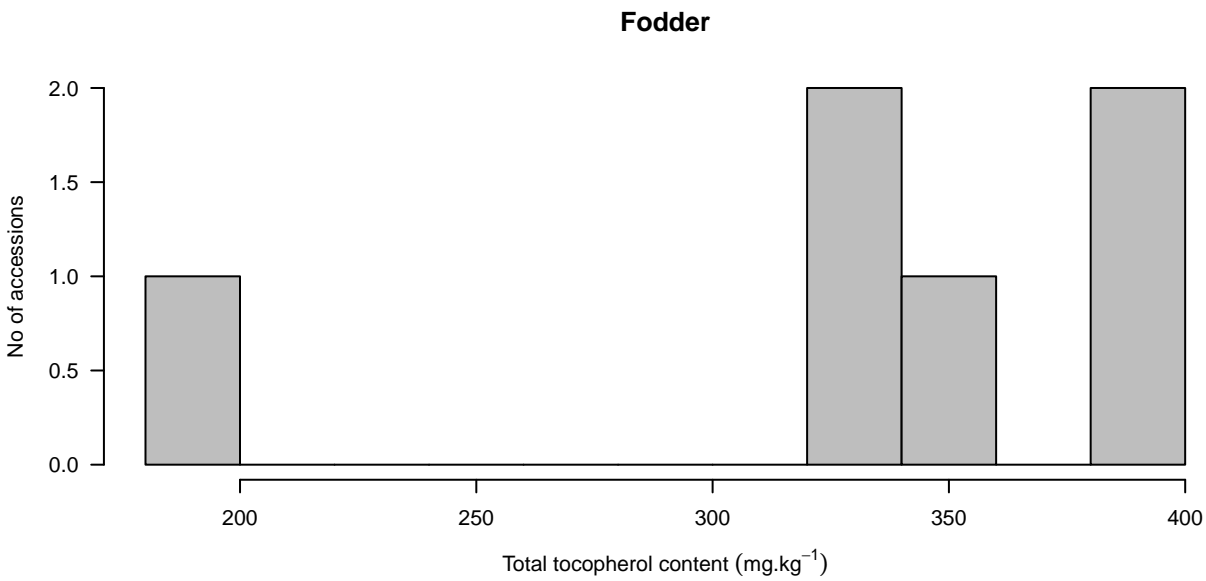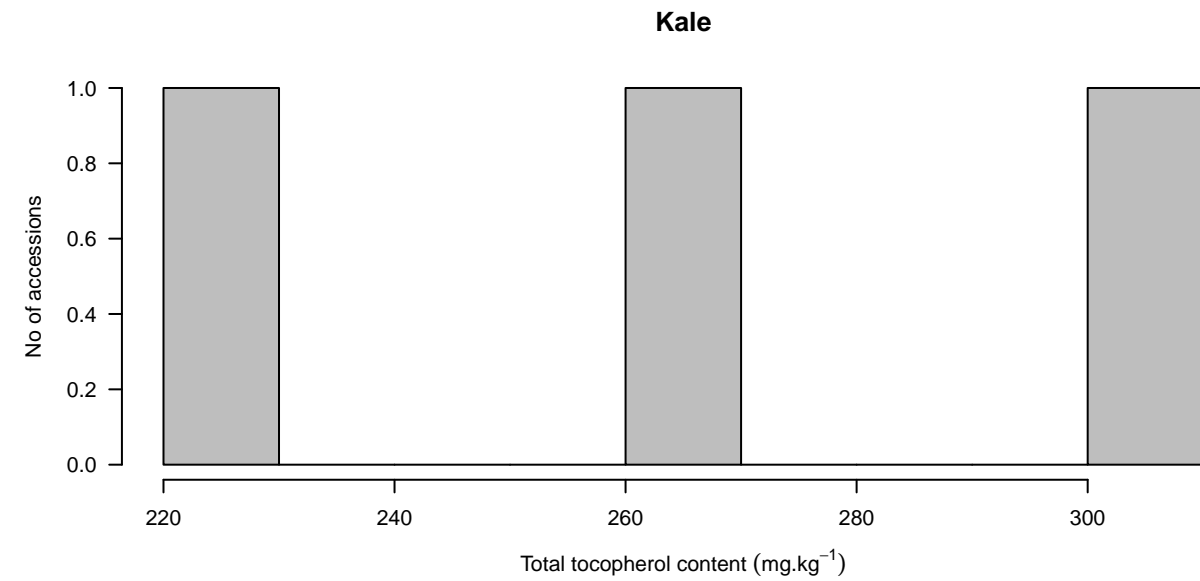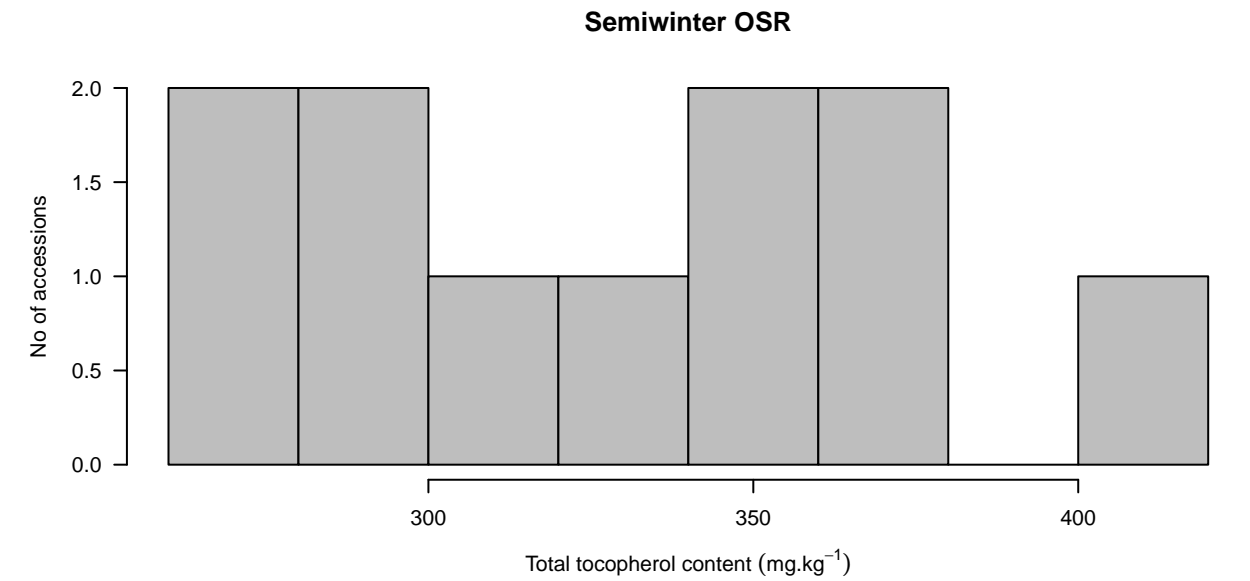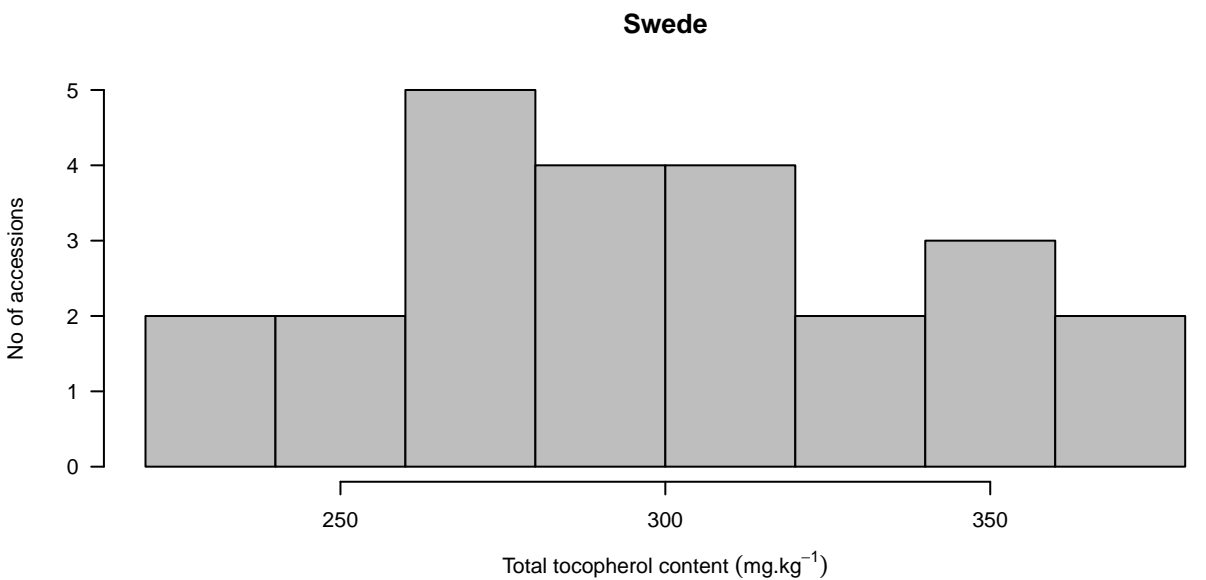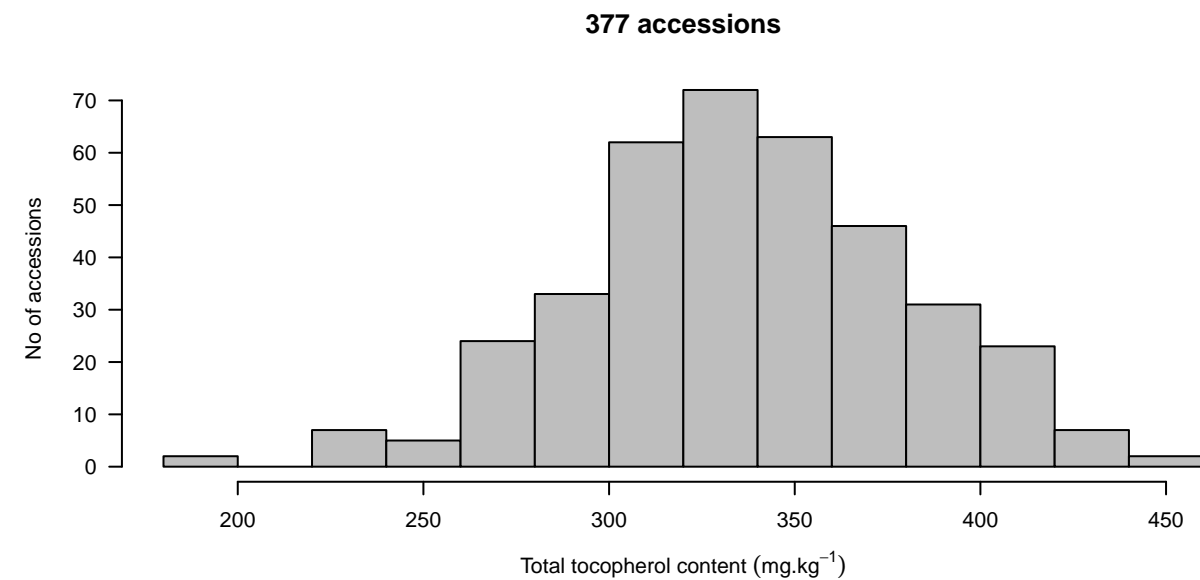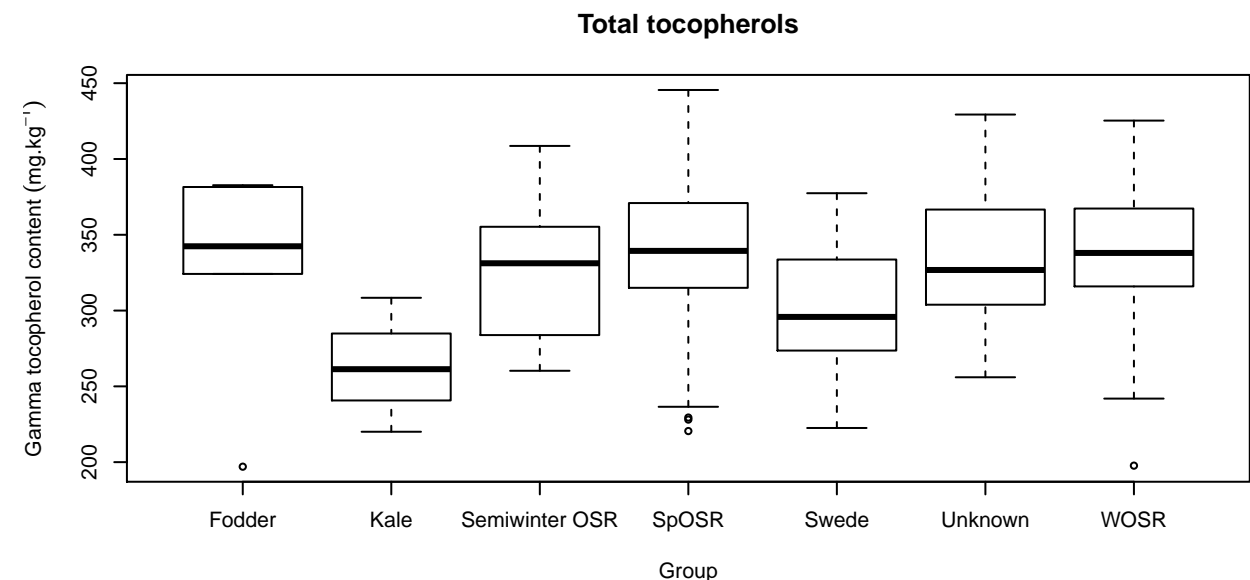

Supplement: Supplementary file 2 — Figure S2. Histograms of seed tocopherol composition of the RIPR diversity panel in different crop types. [file TPJ-93-181-s002.pdf]
