# Supplementary material for: Validation of an updated Associative Transcriptomics platform for the polyploid crop species Brassica napus by dissection of the genetic architecture of erucic acid and tocopherol isoform variation in seeds
Source: Plant J. 2017 Dec 2;93(1):181–92. doi: 10.1111/tpj.13767 (PMC5767744; doi:10.1111/tpj.13767)
Supplement: Supplementary file 3 — Figure S3. Quantile–quantile plots from GEM and SNP association analysis for erucic acid and γ/α‐tocopherol ratio. [file TPJ-93-181-s003.pdf]

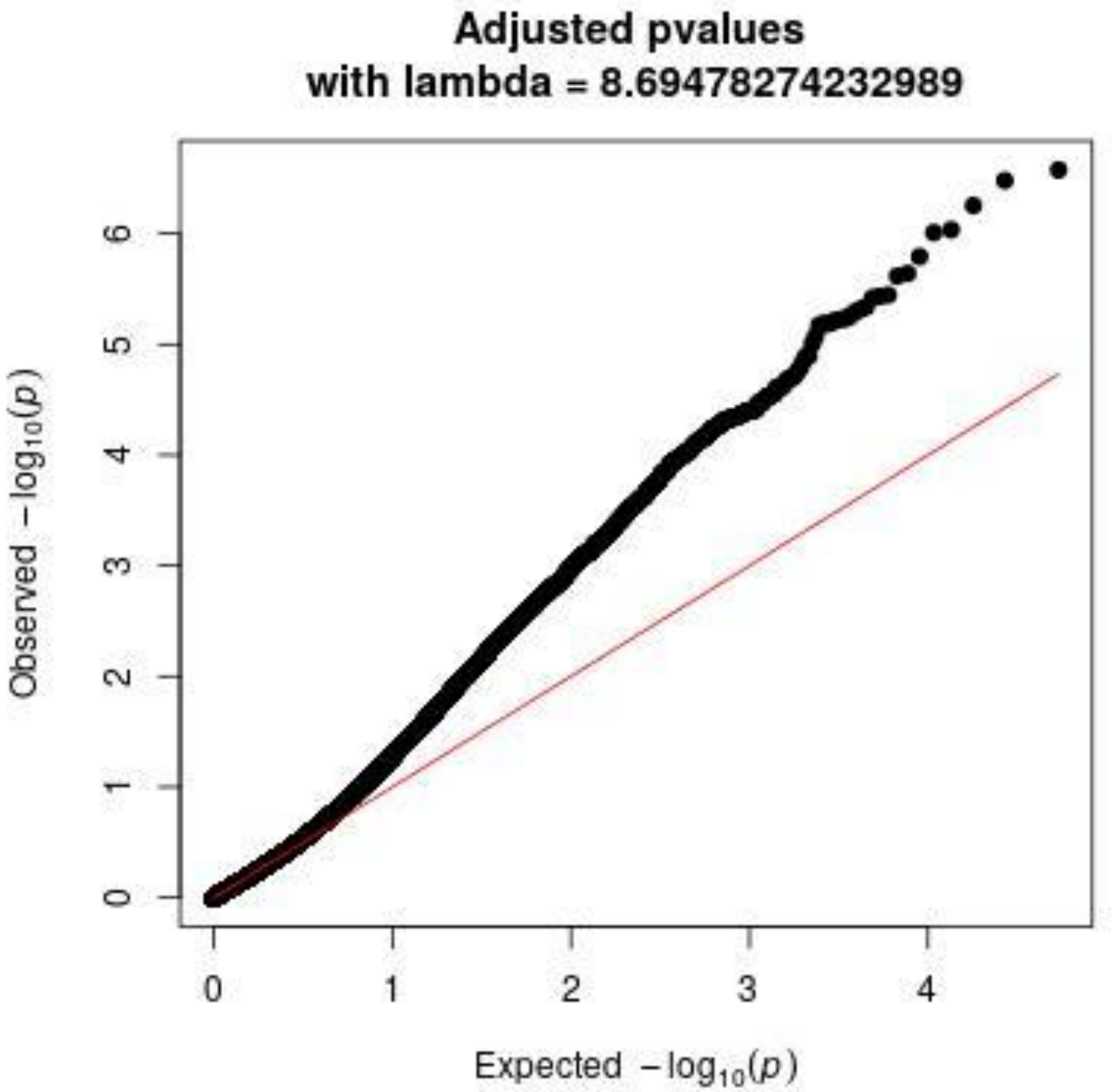

QQ plot from GEM association analysis of γ/α tocopherol ratio

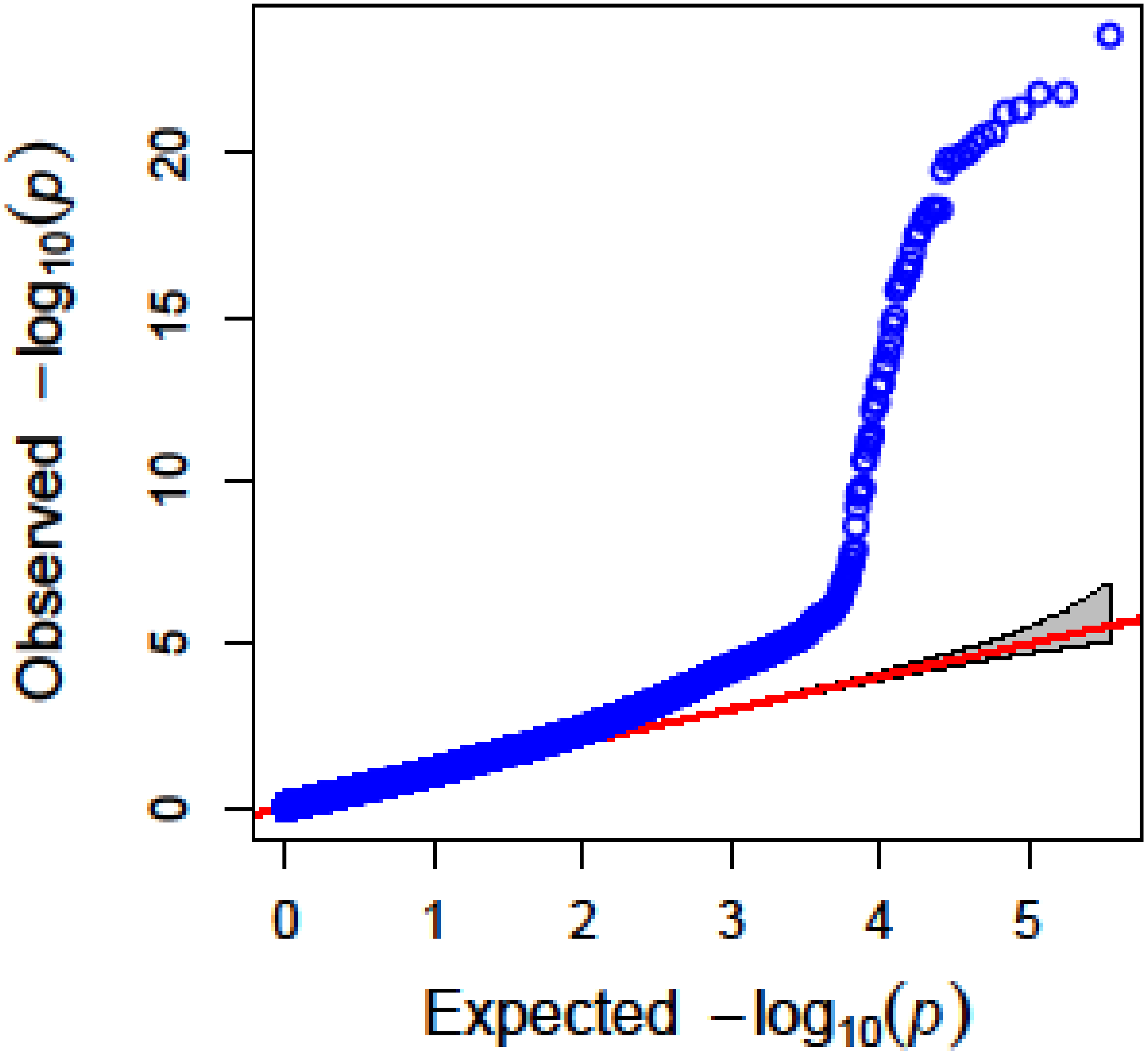

QQ plot from SNP association analysis of γ/α tocopherol ratio

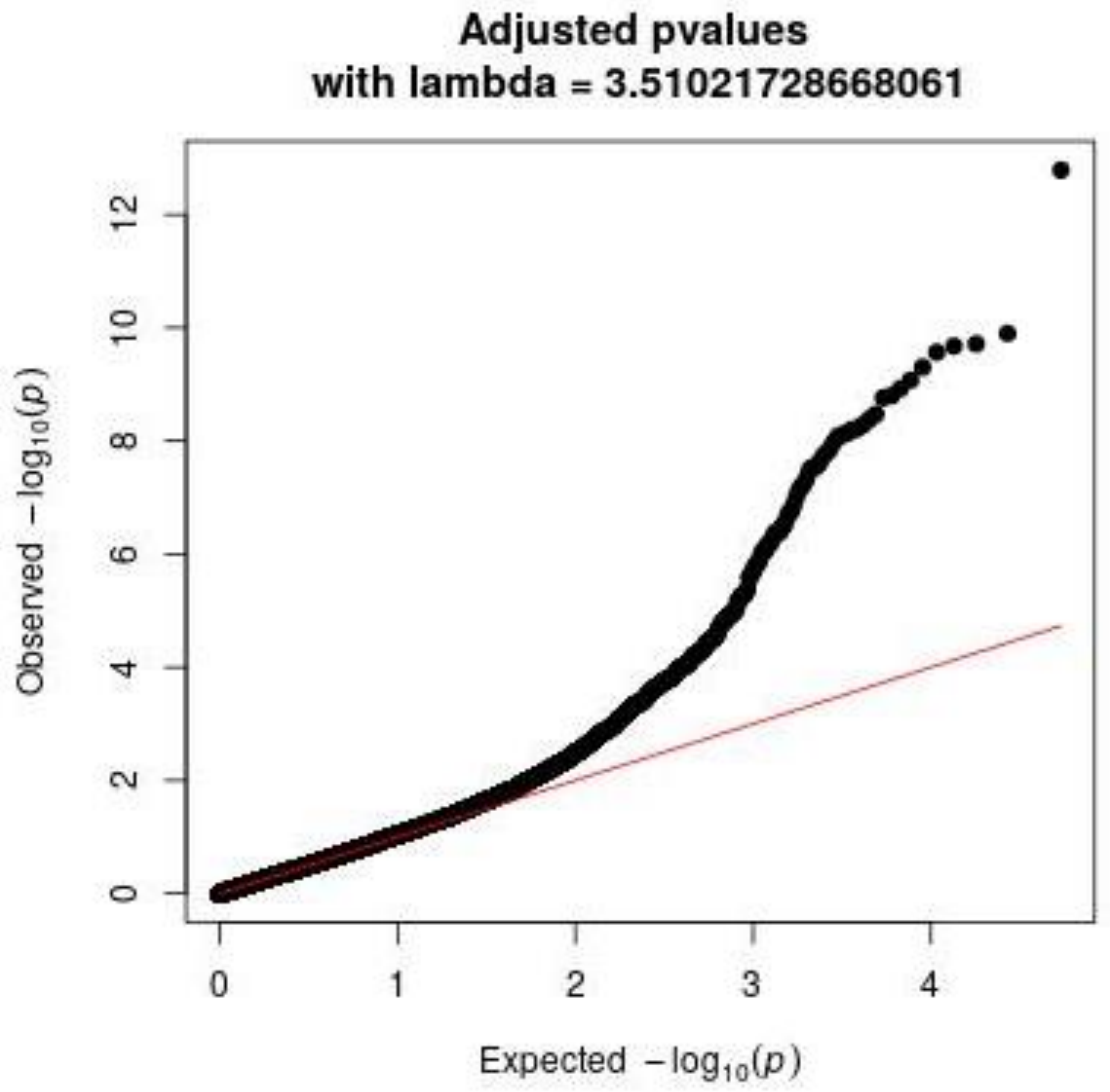

QQ plot from GEM association analysis of erucic acid

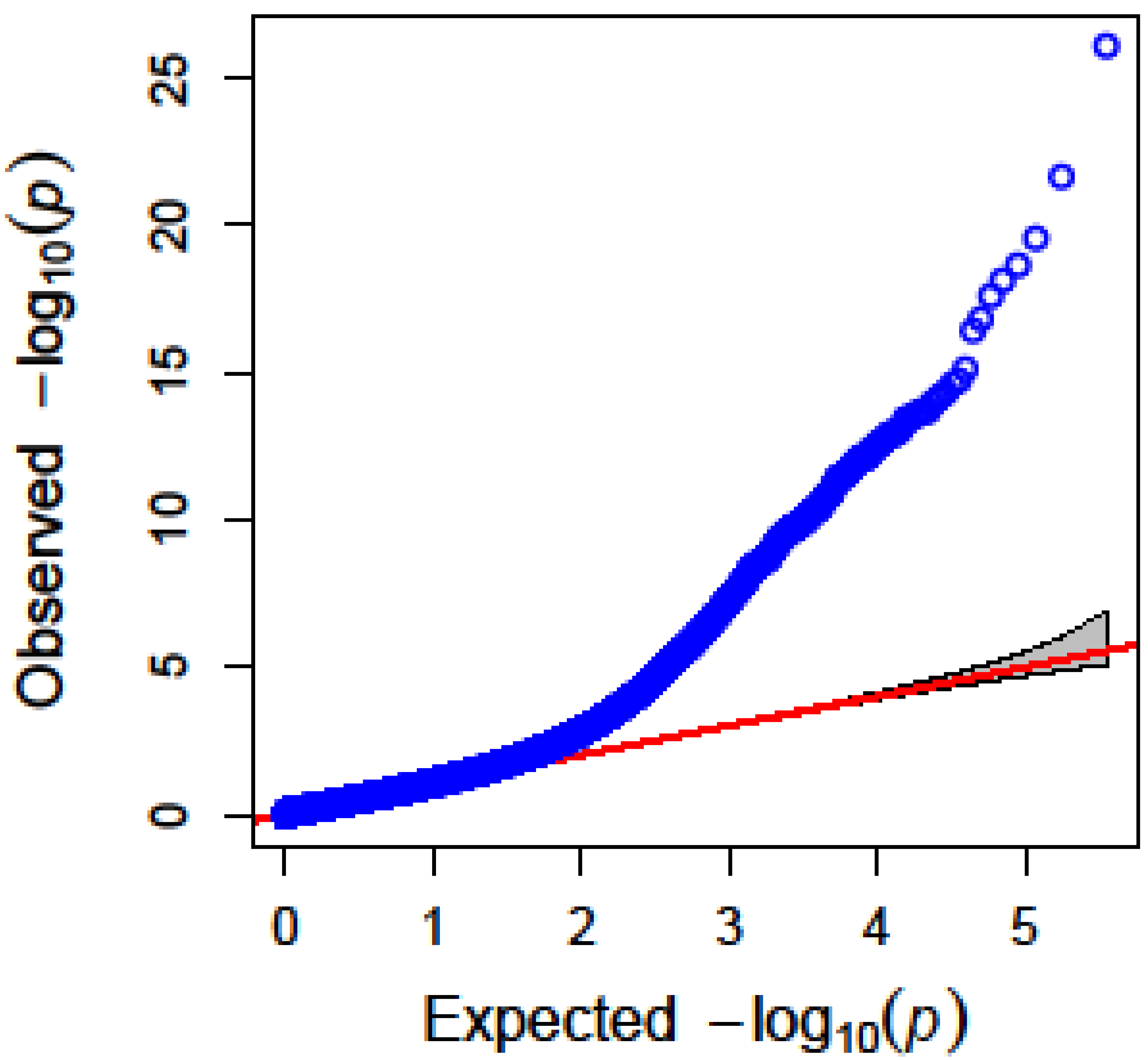

QQ plot from SNP association analysis of erucic acid
