# Supplementary material for: Validation of an updated Associative Transcriptomics platform for the polyploid crop species Brassica napus by dissection of the genetic architecture of erucic acid and tocopherol isoform variation in seeds
Source: Plant J. 2017 Dec 2;93(1):181–92. doi: 10.1111/tpj.13767 (PMC5767744; doi:10.1111/tpj.13767)
Supplement: Supplementary file 11 [file TPJ-93-181-s011.docx]

**Supporting Information Legends**

**Figure S1.** Genome-wide Linkage Disequilibrium analysis for the RIPR diversity panel: Figure S1_LD_SAF_1perc_26-9-17.pdf

**Figure S2.** Histograms of seed tocopherol composition of the RIPR diversity panel in different crop types: Figure S2_histograms of seed tocopherol composition.pdf

**Figure S3.** QQ plots from GEM and SNP association analysis for erucic acid and γ/α tocopherol ratio: Figure S3_QQ_plots.pdf

**Data S1.** List of cultivars, crop type classifications and Illumina read mapping statistics: Data S1_cultivars and read mapping_20-12-16.xlsx.

**Data S2.** Ordered list of CDS gene model-based *Brassica* AC pan transcriptome: Data S2_v11 pan-tanscriptome_20-12-16.xlsx.

**Data S3.** Seed fatty acid composition of the RIPR diversity panel: Data S3_fatty acids_10-04-17.xlsx.

**Data S4.** Markers and genomic regions showing association with variation for erucic acid content: Data S4_erucic-associated regions_30-3-17.xlsx.

**Data S5.** Seed tocopherol composition of the RIPR diversity panel: Data S5_tocopherols_14-10-16.xlsx.

**Data S6.** Markers and genomic regions showing association with variation for γ/α tocopherol ratio: Data S6_tocopherol-associated regions_SNPs.xlsx.

**Data S7.** Gene expression markers showing association with variation for γ/α tocopherol ratio: Data S7_tocopherol-associated regions_GEMs.xlsx
